# Pyruvate Abundance Confounds Aminoglycoside Killing of Multidrug-Resistant Bacteria via Glutathione Metabolism

**DOI:** 10.34133/research.0554

**Published:** 2024-12-18

**Authors:** Jiao Xiang, Si-qi Tian, Shi-wen Wang, Ying-li Liu, Hui Li, Bo Peng

**Affiliations:** ^1^ State Key Laboratory of Bio-Control, Guangdong Province Key Laboratory for Pharmaceutical Functional Genes, School of Life Sciences, Southern Marine Science and Engineering Guangdong Laboratory (Zhuhai), Sun Yat-sen University, Guangzhou 510275, China.; ^2^ Laboratory for Marine Biology and Biotechnology, Marine Fisheries Science and Food Production Processes, Qingdao Marine Science and Technology Center, Qingdao 266237, China.

## Abstract

To explore whether the metabolic state reprogramming approach may be used to explore previously unknown metabolic pathways that contribute to antibiotic resistance, especially those that have been neglected in previous studies, pyruvate reprogramming was performed to reverse the resistance of multidrug-resistant *Edwardsiella tarda*. Surprisingly, we identified a pyruvate-regulated glutathione system that occurs by boosting glycine, serine, and threonine metabolism. Moreover, cysteine and methionine metabolism played a key role in this reversal. This process involved pyruvate-depressed glutathione and pyruvate-promoted glutathione oxidation, which was attributed to the elevated glutathione peroxidase and depressed glutathione reductase that was inhibited by glycine. This regulation inhibited reactive oxygen species (ROS) degradation and thereby elevated ROS to eliminate *E. tarda*. Loss of *metB*, *gpx*, and *gor* of the metabolic pathways increased and decreased resistance, respectively, both in vitro and in vivo, thereby supporting the hypothesis of a pyruvate–cysteine–glutathione system/glycine–ROS metabolic pathway. The role of this metabolic pathway in drug resistance and reprogramming reversal was demonstrated in laboratory-evolved gentamicin-resistant *E. tarda* and other clinically isolated multidrug- and carbapenem-resistant pathogens. Thus, we reveal a less studied antibiotic resistance metabolic pathway along with the mechanisms involved in its reversal.

## Introduction

Antibiotic resistance poses a global challenge to human health and to the animal breeding industry because antibiotic-resistant bacteria mount resistance mechanisms leading to reduced sensitivity to conventional antibiotics [1,2]. As a result, there is an urgent need for novel antibiotics, but drug development pipelines have started to run dry due to limited sources [3,4]. It is necessary, therefore, to find ways to restore/promote the bactericidal efficiency of conventional antibiotics that already suffer from resistance in different bacteria. The classic approach is to first uncover resistance mechanisms and then create new treatments based on those mechanisms. Logically, however, it is possible to first develop new approaches to promote the bactericidal efficiency of the already resistant conventional antibiotics, and then reveal the resistance mechanism(s) based on the mechanism(s) overcome by the newly developed approaches.

The metabolic environment confounds antibiotic sensitivity [5–9]. The recently developed metabolic state/metabolome reprogramming approach provides a green, safe, and effective way to combat antibiotic-resistant pathogens by restoring/promoting conventional antibiotics that already suffer from antimicrobial resistance [10–13]. This approach is based on the hypothesis that the metabolic state is responsible for antibiotic resistance. Antibiotic-sensitive and antibiotic-resistant bacteria have antibiotic-sensitive and antibiotic-resistant metabolic states/metabolomes, respectively; crucial biomarkers between the 2 metabolic states/metabolomes may be exploited to reprogram and reverse the antibiotic resistance. This potentiates conventional antibiotics that suffer from resistance to effectively kill antibiotic-resistant bacteria [14–16]. Recent reports on the metabolic reprogramming approach have indicated that the activated pyruvate cycle (the P cycle) that links the phosphoenolpyruvate (PEP)–pyruvate–acetyl-CoA (AcCoA) pathway to the tricarboxylic acid (TCA) cycle plays a key role in reversing antibiotic resistance by boosting the respiratory chain and purine biosynthesis [17–20]. However, the P cycle acts as a source for many metabolic pathways, which may be involved in the reprogramming. Therefore, metabolic reprogramming by intermediate metabolites such as pyruvate of the P cycle is advantageous for uncovering previously unknown metabolic mechanisms of antibiotic resistance.

Pyruvate is a precursor for some amino acids (alanine, valine, leucine, isoleucine, and lysine) and is also important as the first entry point for many fermentation pathways that reoxidize NAD(P)H [reduced form of nicotinamide adenine dinucleotide phosphate (NADP^+^)]. Pyruvate has 8 metabolic fates: (a) pyruvate dehydrogenase (PDH)-mediated oxidation to acetyl-CoA, (b) oxaloacetate decarboxylase (OXD)-mediated conversion to oxaloacetate, (c) malic enzyme (MAE)-mediated conversion to malate, (d) to acetate by the nonenzymatic generation via H_2_O_2_ or the enzymatic generation via PDH or α-ketoglutarate, (e) lactate dehydrogenase (LDH)-mediated reduction to lactate, (f) alanine amino transferase (ALT)-mediated transamination to alanine [21,22], (g) pyruvateformate-lyase (PFL)-mediated conversion to formate, and (h) cystathionine γ-lyase (CGL)-mediated conversion to cysteine (https://www.kegg.jp/pathway/etr00270) (Fig. S1). Among these fates, PDH-mediated oxidation has been carefully studied in antibiotic resistance [11,14], but the rest are less well studied in antibiotic resistance. Therefore, pyruvate is an ideal model metabolite to reveal previously understudied metabolic pathways of antibiotic resistance based on the metabolic reprogramming strategy.

In this work, pyruvate was used as a metabolic reprogramming metabolite to reverse antibiotic resistance of *Edwardsiella tarda*. *E. tarda* is an intracellular gram-negative pathogen that can infect a broad range of hosts, including humans and fish [23]. *E. tarda* is the first bacterium that is used to test the reversion of antibiotic resistance by the metabolic state reprogramming approach [11]. A pyruvate–cysteine–glutathione system/glycine–reactive oxygen species ROS) metabolic pathway was identified as the most key pyruvate-boosting metabolic pathway against the resistance. Further studies showed that this metabolic pathway was depressed in laboratory-evolved *E. tarda* and clinically isolated multidrug-resistant and/or carbapenem-resistant *Pseudomonas auroginosa*, *Escherichia coli*, *Klebsiella pneumonia*, and methicillin-resistant *Staphylococcus aureus* (MRSA). Consistently, the antibiotic resistance of these pathogens was reversed by exogenous pyruvate, suggesting that the metabolic pathway is involved in antibiotic resistance in bacteria. Therefore, metabolic reprogramming may be an effective approach to reveal antibiotic resistance mechanisms, especially those involved in understudied pathways.

## Results

### Pyruvate potentiates aminoglycosides to effectively kill *E. tarda*

**Fig. 1.**
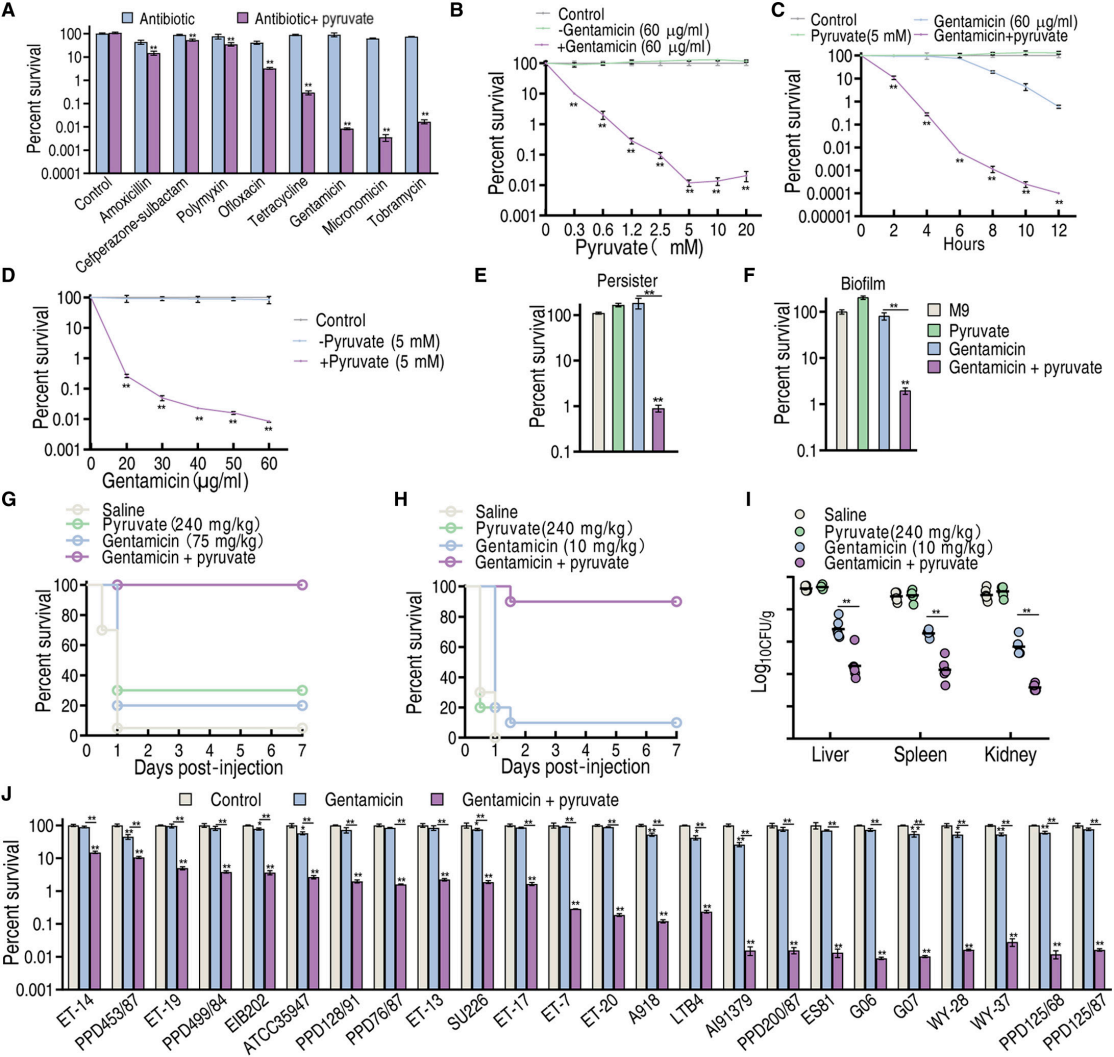
Pyruvate-enabled killing of PPD200/87 by gentamicin. (A) Survival of PPD200/87 (10^8^ CFU/ml) in the synergistic use of 20 mM pyruvate and 100 μg/ml amoxicillin, 200 μg/ml cefperazone-sulbactam, 100 μg/ml polymyxin, 25 μg/ml ofloxacin, 150 μg/ml tetracycline, 50 μg/ml gentamicin, 50 μg/ml micronomicin, or 50 μg/ml tobramycin for 6 h. (B) Survival of PPD200/87 (10^8^ CFU/ml) in the absence or presence of the indicated concentration of pyruvate with and without 60 μg/ml gentamicin. (C) Survival of PPD200/87 (10^8^ CFU/ml) in the indicated incubation time with or without 5 mM pyruvate and/or 60 μg/ml gentamicin. (D) Survival of PPD200/87 (10^8^ CFU/ml) in the indicated dose of gentamicin with or without 5 mM pyruvate for 6 h. (E) Survival of PPD200/87 persisters (10^8^ CFU/ml) in the presence and absence of 5 mM pyruvate and 60 μg/ml gentamicin. (F) Survival of PPD200/87 biofilm (10^8^ CFU/ml) in the presence and absence of 5 mM pyruvate and 60 μg of gentamicin. (G) Survival of tilapia with PPD200/87 (1.1 × 10^7^ CFU) systemic infection in the absence and presence of 240 mg/kg pyruvate, 75 mg/kg gentamicin, or both. (H and I) Survival (H) and organ bacterial number (I) of mice with PPD200/87 (2 × 10^9^ CFU) systemic infection in the absence and presence of pyruvate (10 mg/kg), gentamicin (240 mg/kg), or both. (J) Survival of clinically isolated *E. tarda* strains (10^8^ CFU/ml) in the presence or absence of 5 mM pyruvate and 30 μg/ml gentamicin. Results are displayed as means ± SD, and statistically significant differences are identified by Kruskal–Wallis followed by Dunn’s multiple comparison post hoc test unless otherwise indicated. *P < 0.05 and **P < 0.01.

To explore pyruvate-stimulated antibiotic-mediated killing of *E. tarda*, viability of PPD200/87, a multidrug-resistant *E. tarda* (Fig. S2), was measured in the presence of pyruvate, antibiotics, or both. Five different classes of antibiotics were used, including β-lactams (amoxicillin and cefperazone-sulbactam), polypeptide (polymyxin B), quinolones (ofloxacin), tetracyclines (tetracycline), and aminoglycosides (gentamicin, micronomicin, and tobramycin). Aminoglycosides were most potentiated by pyruvate compared to other classes of antibiotics (Fig. 1A). Further, pyruvate-stimulated gentamicin killing occurred in a pyruvate dose-dependent manner, with 5 mM pyruvate being the most effective (Fig. 1B). Such a synergistic effect was also time and gentamicin dose dependent (Fig. 1C and D). The pyruvate-potentiated gentamicin killing was also effective against persisters and biofilms that could not be eradicated by pyruvate or gentamicin alone (Fig. 1E and F and Fig. S3).

To validate the synergistic effect of pyruvate and gentamicin, 2 infection models were investigated. In the *E. tarda*–tilapia infection model, pyruvate and gentamicin combination treatment elevated the tilapia survival rate from 20% to 100% compared to gentamicin treatment alone (Fig. 1G). In addition, combination treatment promoted mouse survival from 10% to 90% (Fig. 1H). Moreover, pyruvate and gentamicin treatment reduced bacterial loads by 293-, 198-, and 266-fold in mouse liver, spleen, and kidney, respectively (Fig. 1I). Furthermore, over 24 strains of clinically isolated *E. tarda* were tested to exclude strain-specific effects. All strains were more sensitive to killing by pyruvate and gentamicin combination treatment than by monotreatment with either, and the increased killing ranged from 4.2- to 8,130.8-fold (Fig. 1J). These results indicate that pyruvate potentiates aminoglycoside killing of multidrug-resistant *E. tarda*.

### Pyruvate drives metabolic flux into glutathione metabolism

**Fig. 2.**
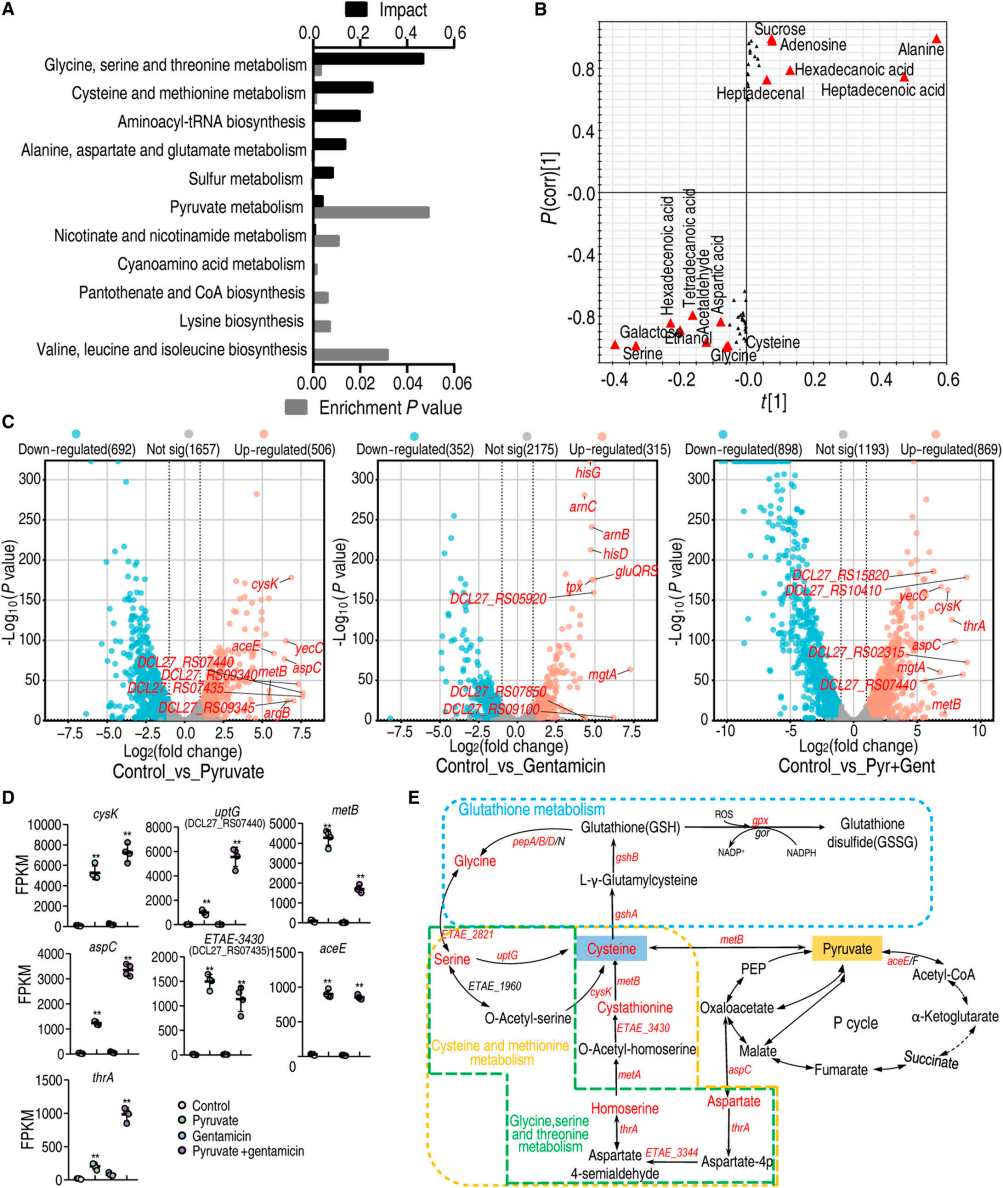
Metabolomic and transcriptional analysis for crucial pathways that are responsible for pyruvate-potentiated gentamicin killing. (A) Metabolic pathways enriched by differentially abundant metabolites. (B) S-plot generated from OPLS-DA illustrates individual metabolites as triangles. Potential biomarkers, indicated in red, are those with an absolute value of covariance *P* ≥ 0.05 and correlation *P*(corr) ≥ 0.5. (C) Volcano plots showing DEGs, where the top 10 up-regulated genes are indicated. (D) Expressional abundance of top 10 genes in glycine, serine, and threonine metabolism; cysteine and methionine metabolism; and glutathione metabolism. (E) Diagram showing that pyruvate fluxes into main metabolic pathways and genes and metabolites in glycine, serine, and threonine metabolism, cysteine and methionine metabolism, and glutathione system. Red, up-regulated gene (italics) and metabolite of glycine, serine, and threonine metabolism; cysteine and methionine metabolism; and glutathione metabolism in metabolomics and transcriptional analysis. Results are displayed as the mean ± SD, and statistically significant differences are identified by Kruskal–Wallis followed by Dunn’s multiple comparison post hoc test unless otherwise indicated. *P < 0.05 and **P < 0.01.

To investigate the mechanism by which pyruvate potentiates gentamicin killing, we performed metabolomic and transcriptomic analyses. Using gas chromatography–mass spectrometry (GC-MS)-based metabolomics, we identified a total of 106 metabolites from each sample (Fig. S4), including 30 up-regulated and 20 down-regulated metabolites (Fig. S5A and B). Pathway analysis indicated that the 50 differentially expressed metabolites were enriched to 11 metabolic pathways, with glycine, serine, and threonine metabolism, as well as cysteine and methionine metabolism being the top 2 pathways (Fig. 2A). Interestingly, the abundance of all and most of the metabolites from the top 1 and 2 enriched metabolic pathways, respectively, was up-regulated (Fig. S5C). Furthermore, orthogonal partial least square discriminant analysis (OPLS-DA) was conducted to identify sample patterns between the 2 groups (Fig. S5D). S-plot analysis identified metabolites with absolute values of the S-plot variation weight *t* and correlation coefficient *P* (corr) ≥ 0.05 and 0.5, respectively, as biomarkers. Among these biomarkers, serine, aspartate, glycine, and cysteine belong to the 2 most impacted metabolic pathways (Fig. 2B and Fig. S5E). Collectively, glycine, serine, and threonine metabolism, as well as cysteine and methionine metabolism are the 2 most critical metabolic pathways boosted by exogenous pyruvate.

Further, RNA-sequencing analysis was performed on bacteria treated with pyruvate, gentamicin, or combination treatment with both. Differentially expressed genes (DEGs) were identified with adjusted *P* values < 0.05 and an absolute |log_2_ fold change| value > 1. In total, 1,198, 667, and 1,767 DEGs were identified in the pyruvate treatment group, gentamicin treatment group, and combination treatment group, respectively. Moreover, 506, 315, and 869 of those were up-regulated, while 692, 352, and 898 were down-regulated in the same groups, respectively (Fig. S6A). All DEGs were visualized using volcano plots, where the top 10 altered genes included *cysk*, *uptG*, *metB*, *aspC*, *ETAE_3430*, and *thrA*, which are implicated in cysteine and methionine metabolism, as well as glycine, serine, and threonine metabolism. Additionally, *aceE*, which plays a role in the P cycle, was also included (Fig. 2C and D and Table S1). Kyoto Encyclopedia of Genes and Genomes (KEGG) pathway analysis of the DEGs also enriched glycine, serine, and threonine metabolism, cysteine and methionine metabolism, and glutathione metabolism (Fig. S6B), rationalizing the shift of these pathways upon combined treatment with pyruvate and gentamicin. Following integration of the metabolomic and transcriptomic data, the 3 pathways were all up-regulated at the transcriptional and metabolite level (Fig. 2E). Pyruvate has 2 pathways (to cysteine by *metB* from pyruvate directly and to aspartate by *aspC* from oxaloacetate indirectly) to flux to cysteine and methionine metabolism, and to glycine, serine, and threonine metabolism, and then possibly to glutathione metabolism. It is important to note that the conversion of pyruvate to oxaloacetate has 3 metabolic pathways (i.e., pyruvate–PEP–oxaloacetate, pyruvate–oxaloacetate, and pyruvate–TCA cycle), which are included in the P cycle (Fig. 2E).

### Exogenous pyruvate boosts glutathione metabolism via glycine, serine, and threonine metabolism and cysteine and methionine metabolism

**Fig. 3.**
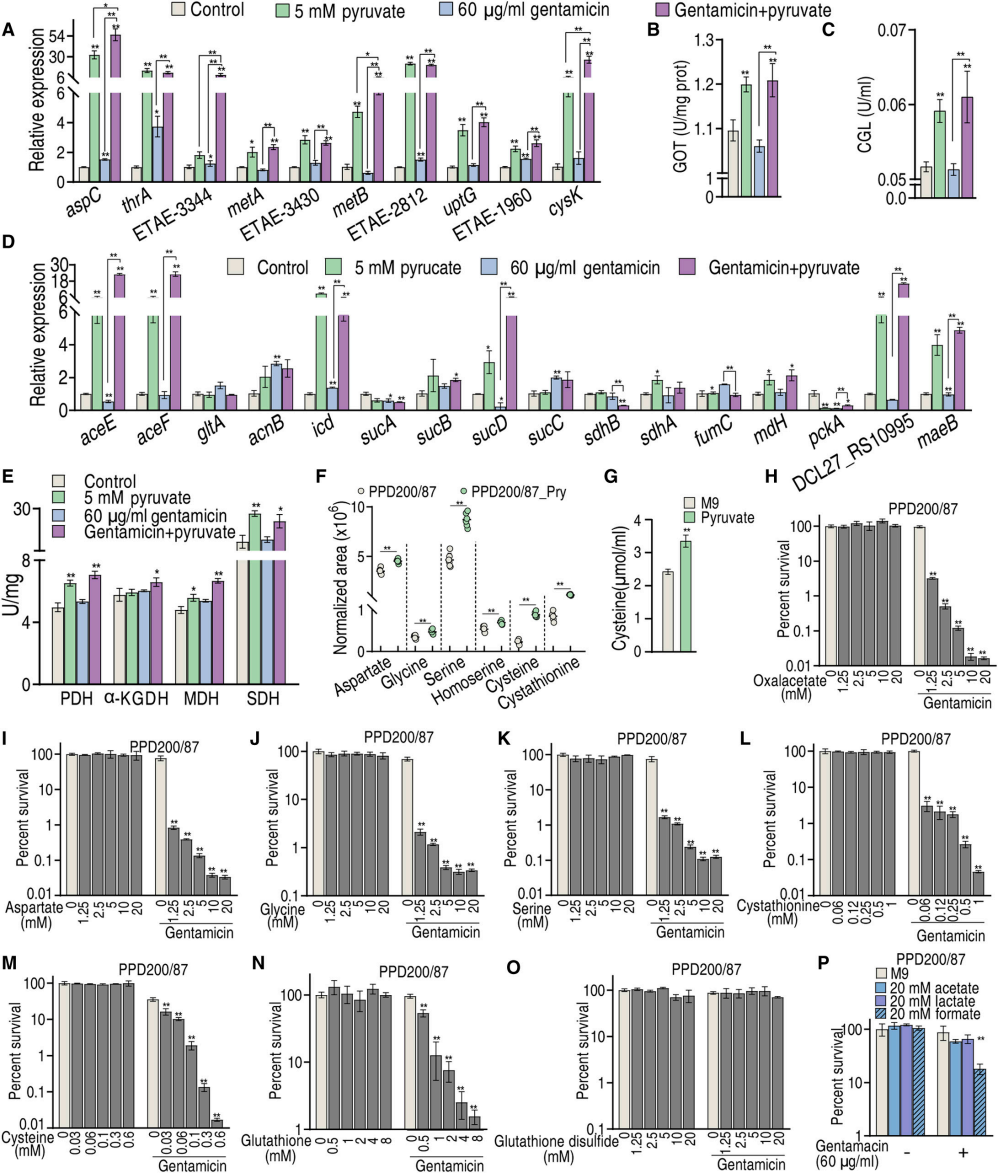
Exogenous pyruvate boosts glutathione metabolism via glycine, serine, and threonine metabolism and cysteine and methionine metabolism. (A) qRT-PCR for expression of genes encoding glycine, serine, and threonine metabolism and cysteine and methionine metabolism in the absence or presence of 5 mM pyruvate and/or 60 μg/ml gentamicin. (B and C) Activity of GOT (B) and CGL (C) in the absence or presence of 5 mM pyruvate and/or 60 μg/ml gentamicin. (D) qRT-PCR for expression of genes encoding the P cycle in the absence or presence of 5 mM pyruvate and/or 60 μg/ml gentamicin. (E) Activity of enzymes of the P cycle in the absence or presence of 5 mM pyruvate and/or 60 μg/ml gentamicin. (F) Abundance of some intermetabolites in glycine, serine, and threonine metabolism, cysteine and methionine metabolism, and glutathione system, quantified by GC-MS. (G) Intracellular level of cysteine in the presence of 5 mM pyruvate in PPD200/87. (H to O) Survival of PPD200/87 (10^8^ CFU/ml) in M9 medium without Mg^++^ and Ca^++^ in the presence of oxaloacetate (H) and aspartate (I) of pyruvate to oxaloacetate, glycine (J) and serine (K) of glycine, serine, and threonine, cysteine (L) and cystathione (M) of cysteine and methionine metabolism, and GSH (N) and GSSG (O) of glutathione plus 60 μg/ml gentamicin. (P) Survival of PPD200/87 (10^8^ CFU/ml) in the presence of formate, acetate, or lactic acid with 60 μg/ml gentamicin. Results are displayed as the mean ± SD, and statistically significant differences are identified by Kruskal–Wallis followed by Dunn’s multiple comparison post hoc test unless otherwise indicated. *P < 0.05 and **P < 0.01.

Thus, quantitative real-time polymerase chain reaction (qRT-PCR) was used to measure expression of genes encoding the 3 metabolic pathways (glycine, serine, and threonine metabolism; cysteine and methionine metabolism; and the P cycle on a holistic basis to fully understand this potentiation). For glycine, serine, and threonine metabolism, as well as cysteine and methionine metabolism, the expression of 10 genes was measured. We found that pyruvate increased the expression of all genes and gentamicin elevated the expression of 4 genes (*aspC*, *thrA*, *ETAE_2812*, and *ETAE_1960*), while the other 6 were unchanged. Distinctly higher expression was detected in the pyruvate group compared to the gentamicin group. More importantly, the synergistic use of pyruvate and gentamicin caused all genes to exhibit high expression compared to the control, where higher expression for most genes was found in the combined therapy treatment group compared to the pyruvate group (Fig. 3A). The enzymes, glutamic-oxaloacetic transaminase (GOT) and CGL, by which pyruvate enters the 2 pathways were also measured. GOT (encoded by *aspC*) reversibly converts oxaloacetate and glutamate into aspartate and α-ketoglutarate, while CGL (encoded by *metB*) reversibly converts pyruvate to cysteine. We observed increased GOT and CGL activity in the pyruvate treatment group as well as in the combination treatment group compared to the control (Fig. 3B and C). For the P cycle, pyruvate mono- and combination treatment promoted the expression of *ace/F*, *icd*, *sucD*, DCL27_RS10995, and *maeB*, but inhibited the expression of *sucA*, *sdhB* (both only the combination treatment group), and *pckA*. Gentamicin inhibited the expression of *aceE*, *sucA*, *sucD*, and *pckA* and promoted the expression of *acnB*, *icd*, and *sucC* (Fig. 3D). The activated P cycle was demonstrated by increased activity of enzymes in the P cycle (Fig. 3E). Furthermore, pyruvate was replaced with one of the intermediate metabolites in glycine, serine, and threonine metabolism; cysteine and methionine metabolism; and glutathione metabolism to determine whether they play a similar role to pyruvate. To achieve this, several intermediate metabolites, including aspartate, glycine, serine, homoserine, cysteine, and cystathionine, were quantified. All were higher in PPD200/87 in the presence than absence of pyruvate (Fig. 3F). Since sulfur-containing metabolites are sensitive to oxygen and heat, we directly quantified l-cysteine level to exclude such effect during GC-MS sample preparation [24]. This test validates that pyruvate increased cysteine content (Fig. 3G). Intermediate metabolites of the pyruvate to glutathione metabolism were individually synergized with gentamicin to kill PPD200/87, including oxaloacetate, aspartate, glycine, serine, cystathionine, cysteine, glutathione (GSH), and glutathione disulfide (GSSG). All of these metabolites synergized with gentamicin to kill *E. tarda* in a dose-dependent manner, with the exception of GSSG, which had no effect (Fig. 3H to O). GSH and GSSG are the principal intracellular antioxidant buffers against oxidative stress. GSH was the end effector, implying that the pyruvate-potentiated killing is related to GSH and the GSH/GSSG ratio. In addition, we tested the role of acetate, lactate, and formate, 3 products of other pyruvate metabolic pathways, in potentiating gentamicin. Formate, but not acetate and lactate, weakly potentiated the gentamicin killing (Fig. 3P), suggesting that pyruvate does not transform these 3 products to play the key role. These findings not only support the hypothesis that a pyruvate–cysteine/oxaloacetate–aspartate–serine–cysteine–glutathione pathway contributes to the pyruvate potentiation but also indicate the importance of understanding the balance between GSH and GSSG.

### Exogenous pyruvate regulates redox to promote gentamicin killing

**Fig. 4.**
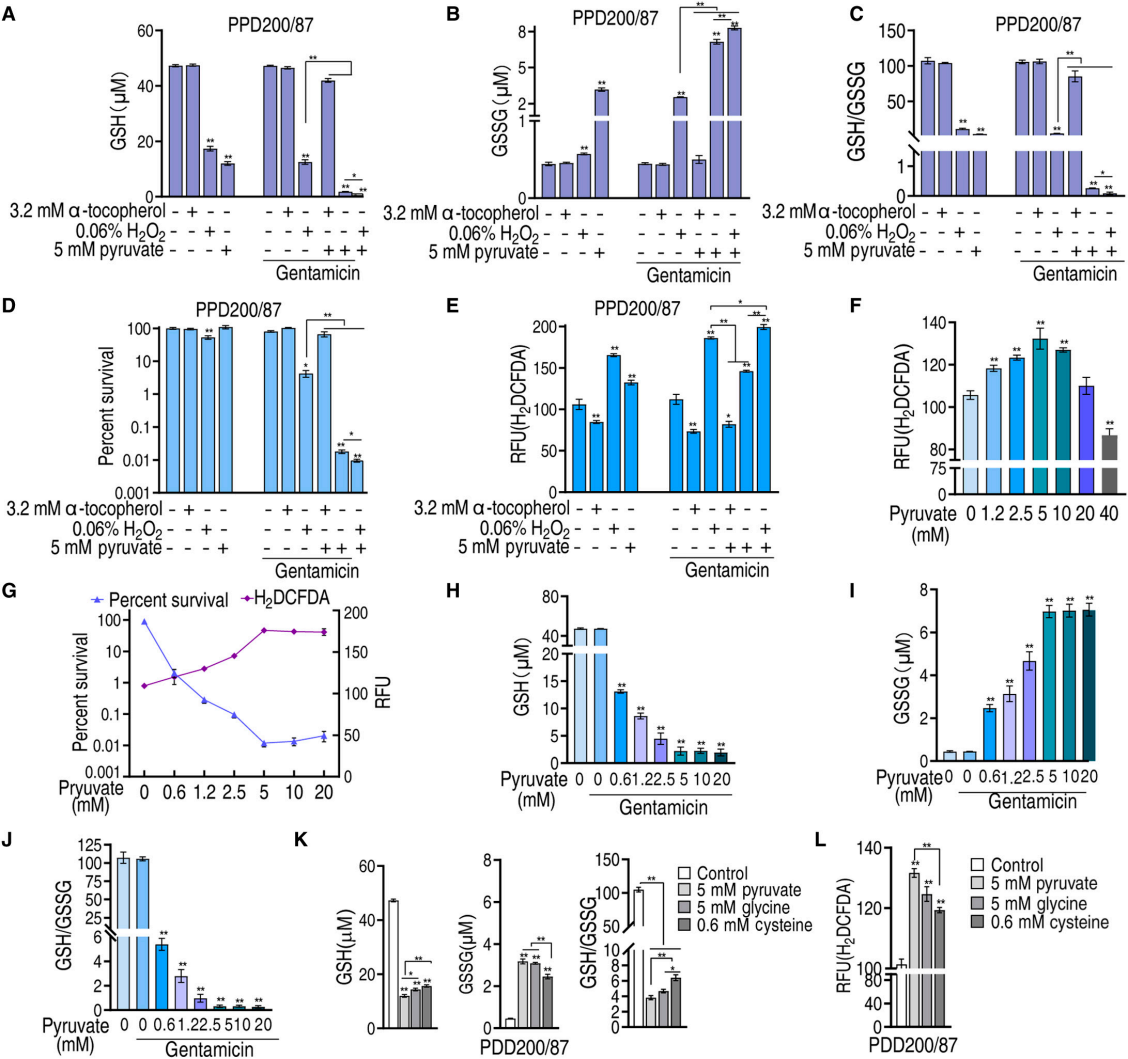
Redox modulated by exogenous pyruvate affects gentamicin killing. (A) GSH of PPD200/87 in the absence or presence of 5 mM pyruvate and/or 60 μg/ml gentamicin plus 3.2 mM α-tocopherol, or 0.06% H_2_O_2_. (B) GSSG of PPD200/87 in the absence or presence of 5 mM pyruvate and/or 60 μg/ml gentamicin plus 3.2 mM α-tocopherol, or 0.06% H_2_O_2_. (C) Ratio of GSH/GSSG in (A) and (B). (D) Survival of PPD200/87 (10^8^ CFU/ml) in the presence of 60 μg/ml gentamicin and 5 mM pyruvate plus 3.2 mM α-tocopherol, or 0.06% H_2_O_2_. (E) ROS content in (D). (F) ROS of PPD200/87 in the indicated concentration of pyruvate. (G) Relationship between survival and ROS of PPD200/87 (10^7^ CFU) in the presence of the indicated concentrations of pyruvate and 60 μg/ml gentamicin. (H) GSH of PPD200/87 in the indicated concentration of pyruvate and 60 μg/ml gentamicin. (I) GSSG of PPD200/87 in the indicated concentration of pyruvate and 60 μg/ml gentamicin. (J) GSH/GSSG in (H) and (I). (K) GSH, GSSG, and their ratio of PPD200/87 in the absence or presence of 5 mM pyruvate, 5 mM glycine, or 0.6 mM cysteine. (L) ROS of PPD200/87 (10^7^ CFU) in the absence or presence of 5 mM pyruvate, 5 mM glycine, or 0.6 mM cysteine. Results are displayed as the mean ± SD, and statistically significant differences are identified by Kruskal–Wallis followed by Dunn’s multiple comparison post hoc test unless otherwise indicated. *P < 0.05 and **P < 0.01.

Cysteine and methionine metabolism and glutathione metabolism provide sulfur-containing amino acids and low molecular weight thiol compounds, respectively, which serve as potent antioxidants and redox signaling intermediates [25]. In glutathione metabolism, 2 molecules of GSH are oxidized by GSH peroxidase to one molecule of GSSG to directly scavenge ROS [26], while glutathione reductase (GR) converts GSSG to 2 molecules of GSH primarily using nicotinamide adenine dinucleotide phosphate (NADPH) or, in rare cases, NADH [reduced form of nicotinamide adenine dinucleotide (oxidized form) (NAD^+^)] [27]. Thus, GSH and GSSG and their ratio were measured in the absence and presence of pyruvate and/or gentamicin, in addition to the ROS scavenger, α-tocopherol, and the ROS promoter, H_2_O_2_ for positive and negative controls, respectively. The GSH level was reduced with the addition of pyruvate or H_2_O_2_, but not of α-tocopherol. In the combination treatment with gentamicin, GSH levels were considerably inhibited by pyruvate and then H_2_O_2_, but weakly by α-tocopherol. The action of H_2_O_2_ and α-tocopherol was enhanced by pyruvate (Fig. 4A). In contrast, we observed the opposite with GSSG levels (i.e., GSSG level was elevated with addition of pyruvate or H_2_O_2_). In the combination treatment with gentamicin, the GSSG level was greatly increased by pyruvate and then H_2_O_2_, which was enhanced by pyruvate. Importantly, α-tocopherol did not influence these effects (Fig. 4B). The GSH/GSSG ratio showed that pyruvate and H_2_O_2_ decreased the ratio, especially in the synergy with gentamicin and pyruvate, whereas the ROS scavenger and gentamicin treatment alone did not affect the ratio (Fig. 4C). Consistently, the second and first lowest viability was detected with the synergistic use of gentamicin and pyruvate, as well as with the addition of H_2_O_2_, respectively. Higher and lower viability was detected in the gentamicin/α-tocopherol combination treatment group and with H_2_O_2_, respectively, compared to gentamicin alone. Unchanged survival was measured in addition to α-tocopherol and gentamicin with and without pyruvate (Fig. 4D). The viability should be attributed to ROS. Specifically, α-tocopherol inhibited ROS, while H_2_O_2_ and pyruvate stimulated ROS, which was enhanced by gentamicin and pyruvate (Fig. 4E). Further, low concentrations of pyruvate promoted ROS, while high concentrations inhibited ROS in the absence of gentamicin (Fig. 4F), which is consistent with previous studies of pyruvate as a ROS scavenger [28]. Pyruvate-mediated ROS enhancement is positively proportional to pyruvate-mediated gentamicin killing within 5 mM pyruvate, but parallel when >5 mM pyruvate is used (Fig. 4G). These results indicate that pyruvate regulates GSH, GSSG, and GSH/GSSG ratios to promote ROS, which is needed for pyruvate-potentiated gentamicin killing. To further confirm this, GSH, GSSG, and GSH/GSSG ratios were measured in the presence of gentamicin with a pyruvate concentration gradient. Indeed, GSH and GSSG were reduced and elevated with the increasing pyruvate, respectively, and reached their maxima at approximately 5 mM pyruvate (Fig. 4H and I), which is a similar concentration observed to potentiate gentamicin killing (Fig. 1B). Consistently, we found that the GSH/GSSG ratio decreased in a dose-dependent manner (Fig. 4J). GSH, GSSG, GSH/GSSG ratio, and ROS were also measured in the presence of glycine and cysteine, the downstream metabolites of pyruvate. As compared to pyruvate (5 mM), which decreased GSH for 3.94-fold, increased GSSG for 7.17-fold, and reduced GSH/GSSG for 28.13-fold (Fig. 4A to C), glycine (5 mM) and cysteine (0.6 mM) decreased GSH level for 3.28- and 3.01-fold, increased GSSG for 6.82- and 5.42-fold, and decreased GSH/GSSG ratio for 22.43- and 16.34-fold, respectively (Fig. 4K). The folds of change for glycine and pyruvate were similar to pyruvate, but cysteine was less potent, which can be due to the lower dose of cysteine we used because of its poor solubility in water. Similarly, ROS was 121.3 ± 4.2 relative fluorescence units (RFU) and 110.7 ± 1.5 RFU in the presence of glycine and cysteine, respectively, as compared to 132.3 ± 4.9 RFU in the presence of pyruvate (Fig. 4F and L). Taken together, these experiments showed that pyruvate reaches its optimum effect at 5 mM. Moreover, these data suggest that regulation of GSH and GSSG ratio by exogenous pyruvate is responsible for ROS abundance and viability.

### Mechanism by which pyruvate regulates glutathione system and relationship between gentamicin killing and ROS

**Fig. 5..**
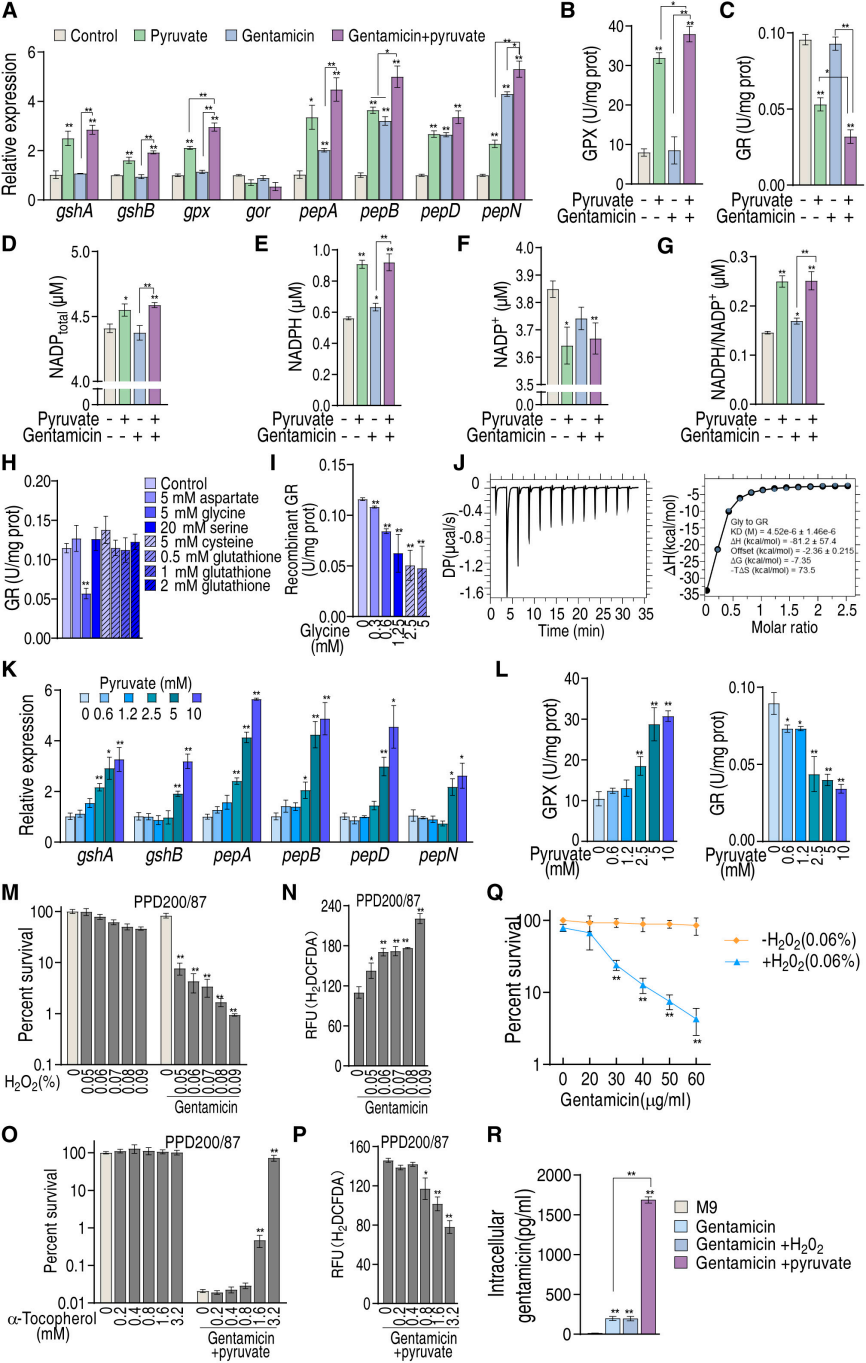
Glutathione metabolism and relationship between ROS and gentamicin killing. (A) qRT-PCR for expression of genes encoding glutathione metabolism in PPD200/87. (B and C) Activity of GPX (B) and GR (C) of PPD200/87 in the absence or presence of 60 μg/ml gentamicin and/or 5 mM pyruvate. (D to G) NADP_total_ (D), NADPH (E), NADP^+^ (F), and NADPH/NADP^+^ (G) of PPD200/80 in the absence or presence of 60 μg/ml gentamicin and/or 5 mM pyruvate. (H) Activity of GR in the presence of the indicated concentrations of the indicated metabolites. (I) Activity of recombinant GR in the presence of the indicated concentrations of glycine. (J) ITC for identifying the binding of glycine with recombinant GR. (K) qRT-PCR for expression of genes encoding cysteine to GSH and GSH to glycine in the presence of the indicated concentrations of pyruvate. (L) Activity of GPX and GR in the presence of the indicated concentrations of pyruvate. (M) Survival of PPD200/87(10^8^ CFU/ml) in the presence of the indicated concentration of H_2_O_2_ plus 60 μg/ml gentamicin. (N) ROS of PPD200/87 in the presence of the indicated concentration of H_2_O_2_ plus 60 μg/ml gentamicin. (O) Survival of PPD200/87 (10^8^ CFU/ml) in the presence of the indicated concentration of α-tocopherol plus 60 μg/ml gentamicin and 5 mM pyruvate. (P) ROS of PPD200/87 in the presence of the indicated concentration of α-tocopherol plus 60 μg/ml gentamicin and 5 mM pyruvate. (Q) Survival of PPD200/87 in the presence of H_2_O_2_ and the indicated concentrations of gentamicin. (R) Intracellular gentamicin of PPD200/87 in the presence of pyruvate or H_2_O_2_. Results are displayed as the mean ± SD, and statistically significant differences are identified by Kruskal–Wallis followed by Dunn’s multiple comparison post hoc test unless otherwise indicated. *P < 0.05 and **P < 0.01.

To further examine the pyruvate-mediated glutathione system regulation, qRT-PCR was used to measure the expression of genes encoding GSH generation and conversion. The expression of *gshA* and *gshB*, which convert cysteine and glycine to GSH, was higher in pyruvate and pyruvate/gentamicin combination treatment groups compared to the control and gentamicin monotreatment groups. However, the expression of genes that convert GSH to glycine (i.e., *pepA*, *pepB*, *pepD*, and *pepN*) was higher in the pyruvate, gentamicin, and pyruvate/gentamicin combination treatment groups compared to the control, with the highest expression observed in the combination group. Very interestingly, a reversal change was determined between higher *gpx* and low *gor* (no significance) expression in the pyruvate and pyruvate/gentamicin groups compared to the gentamicin only and control groups (Fig. 5A and Fig. S7). Note that *gpx* encodes glutathione peroxidase (GPX) that oxidizes GSH to GSSG, while *gor* encodes GR, which reduces GSSG back to GSH. Similar results (pyruvate caused the higher GPX activity and the lower GR activity, which were enhanced by gentamicin) were measured for the activity of GPX and GR (Fig. 5B and C). These results suggest that the reduction of GSSG to GSH is impaired by combination treatment of pyruvate and gentamicin. Since NADPH is required for reduction, NADP_total_, NADPH, NADP^+^, and the NADHP/NADP^+^ ratio were measured. Pyruvate and pyruvate with gentamicin elevated NADP, NADPH, and reduced NADP^+^, leading to an increased NADHP/NADP^+^ ratio (Fig. 5D to G). These results indicate that the depressed GR is the most characteristic feature in the synergistic use of pyruvate and gentamicin, which is not related to the NADHP/NADP^+^ ratio. Because exogenous pyruvate alone caused a similar effect to the activity of GR, we speculated that pyruvate or its conversion products inhibited the activity of GR directly. To explore this, the GR activity was measured in the presence of aspartate, glycine, serine, cysteine, GSH, or GSSG. The activity was inhibited by glycine but not by the other metabolites (Fig. 5H). To validate these data, *gor* was cloned and the expressed recombinant protein was used to measure its activity in the presence of different concentrations of glycine. Our data show that its activity was reduced with increasing concentrations of glycine (Fig. 5I). Further, isothermal titration calorimetry (ITC) analysis revealed the binding of glycine with GR (Fig. 5J).

To further explain the relationship between pyruvate-boosted cysteine and pyruvate-depressed GSH, the expression of genes encoding the conversion from cysteine to GSH and from GSH to glycine was measured and the activity of GPX and GR was detected in the presence of different concentrations of pyruvate. The expression of these genes and GPX activity were elevated with increasing concentrations of pyruvate (Fig. 5K and L), while GR activity was reduced in a pyruvate dose-dependent manner (Fig. 5L). These results suggest that pyruvate metabolic flux promotes GSH and glycine, and subsequently, glycine in turn inhibits GR to depress the GSH source from GSSG, thereby resulting in GSH depression.

We further supposed that the depressed GSH impairs the clearance of ROS and thereby caused ROS elevation, which is responsible for the pyruvate-potentiated gentamicin killing. Thus, pyruvate was replaced with H_2_O_2_ to test whether H_2_O_2_ potentiated gentamicin killing. We found that the viability of PPD200/87 was reduced in a H_2_O_2_ dose-dependent manner (Fig. 5M), while ROS was elevated with the increasing H_2_O_2_ concentrations (Fig. 5N). When α-tocopherol was added, the viability and ROS were up- and down-regulated, respectively, in an α-tocopherol dose-dependent manner (Fig. 5O and P). Viability was also related to gentamicin dose under the same level of H_2_O_2_, the dose that had no killing effect (Fig. 5Q). To understand why pyruvate and H_2_O_2_ caused the elevated ROS but relatively higher and lower killing with the same dose of gentamicin, intracellular drug concentration was measured. Pyruvate elevated drug intracellular concentration, but H_2_O_2_ did not (Fig. 5R). These results support the conclusion that pyruvate regulates glutathione system to accumulate ROS that potentiates gentamicin killing and that gentamicin killing is ROS dependent. Therefore, the pyruvate–cysteine–glutathione system/glycine–ROS metabolic pathway is potentiated to synergize gentamicin killing.

### Absence of *metB*, *aspC*, *gpx*, *gor*, or *pepB* confers antibiotic resistance

**Fig. 6.**
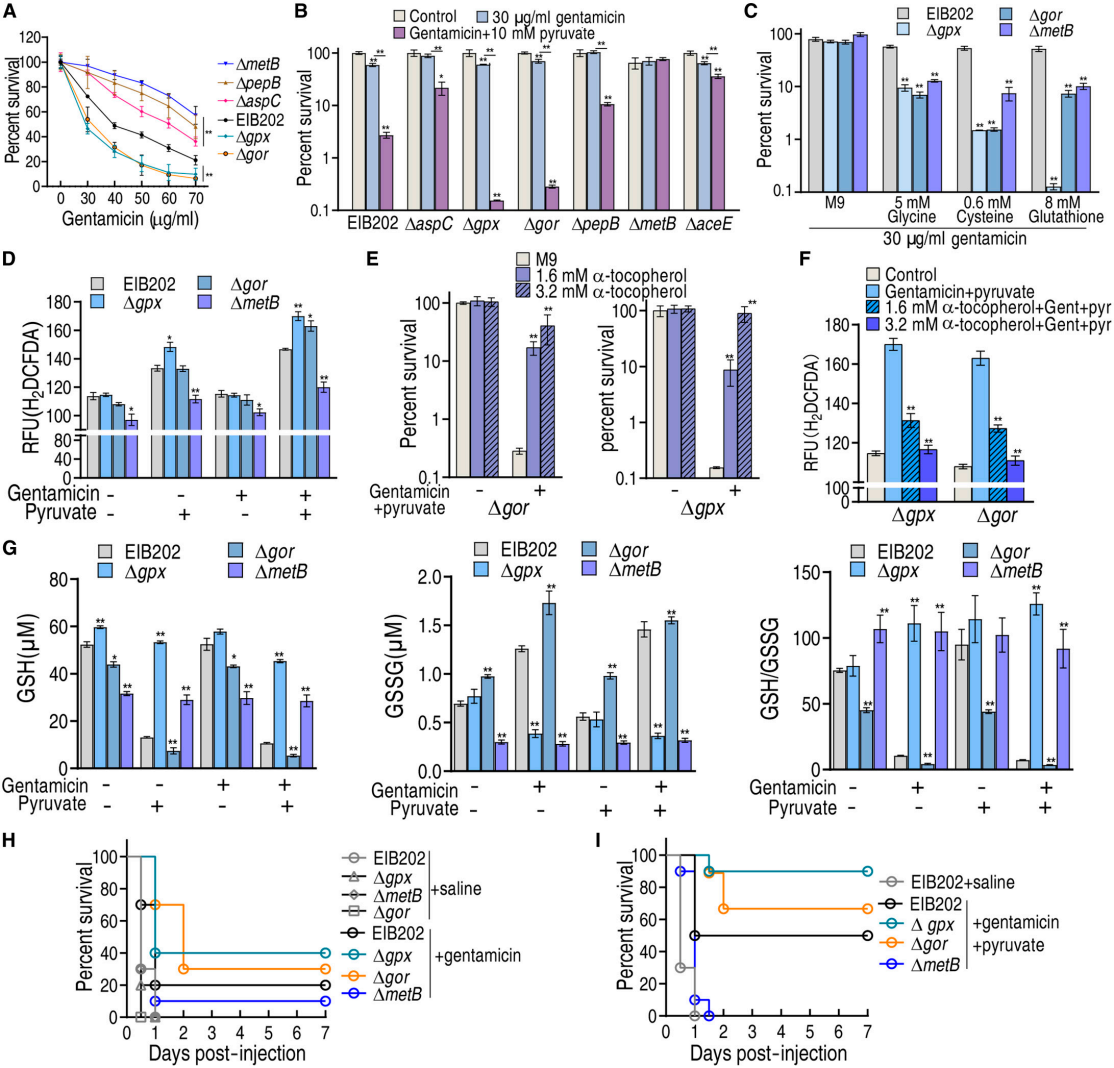
Physiological validation of pyruvate–aspartate–glycine–glutathione–ROS pathway. (A) Percent survival of EIB202 (10^8^ CFU/ml) and its mutants Δ*metB* (10^8^ CFU/ml), Δ*aspC* (10^8^ CFU/ml), Δ*pepB* (10^8^ CFU/ml), Δ*gor* (10^8^ CFU/ml), and Δ*gpx* (10^8^ CFU/ml) in the indicated dose of gentamicin. (B) Percent survival of EIB202 (10^8^ CFU/ml) and its mutants Δ*metB* (10^8^ CFU/ml), Δ*aspC* (10^8^ CFU/ml), Δ*pepB* (10^8^ CFU/ml), Δ*gor* (10^8^ CFU/ml), Δ*gpx* (10^8^ CFU/ml), Δ*metB* (10^8^ CFU/ml), and Δ*aceE* (10^8^ CFU/ml) in the absence or presence of 10 mM pyruvate and/or 30 μg/ml gentamicin. (C) Percent survival of EIB202 (10^8^ CFU/ml) and its mutants Δ*metB* (10^8^ CFU/ml), Δ*gor* (10^8^ CFU/ml), and Δ*gpx* (10^8^ CFU/ml) in the absence or presence of 5 mM glycine, 0.625 mM cysteine, or 8 mM glutathione and/or 30 μg/ml gentamicin. (D) ROS of EIB202 and its mutants Δ*metB*, Δ*gor*, and Δ*gpx* (all 10^8^ CFU) in the absence or presence of 10 mM pyruvate and/or 30 μg/ml gentamicin. (E) Percent survival of Δ*gor* (10^8^ CFU/ml) and Δ*gpx* (10^7^ CFU/ml) in the absence or presence of 10 mM pyruvate and 30 μg/ml gentamicin with or without the indicated concentration of α-tocopherol. (F) ROS of Δ*gor* and Δ*gpx* in the presence of 10 mM pyruvate and/or 30 μg/ml gentamicin with or without the indicated concentration of α-tocopherol. (G) GSH and GSSG and their ratio of EIB202 and their mutants Δ*metB*, Δ*gor*, and Δ*gpx* in the absence or presence of 10 mM pyruvate and/or 30 μg/ml gentamicin. (H and I) Survival of mice infected with EIB202 and their mutants Δ*gpx*, Δ*gor*, and Δ*metB* (4 × 10^8^ CFU, each strain). Mice were infected separately with EIB202 and its mutants Δ*metB*, Δ*gpx*, and Δ*gpx* (*n* = 10 per group) and then treated with 10 mg/kg gentamicin (H) plus 120 mg/kg pyruvate (I). Results are displayed as the mean ± SD, and statistically significant differences are identified by Kruskal–Wallis followed by Dunn’s multiple comparison post hoc test unless otherwise indicated. *P < 0.05 and **P < 0.01.

The role of the pyruvate–cysteine–glutathione system/glycine–ROS metabolic pathway in antibiotic resistance was further evaluated using deletion mutants. Loss of *metB*, *aspC*, *pepB*, *gpx*, or *gor* elevated (*metB*, *aspC*, *pepB*) and decreased (*gpx*, *gor*) viability in the presence of gentamicin alone (Fig. 6A), suggesting the physiological existence of this metabolic resistance pathway. When exogenous pyruvate was added, the viability of Δ*aspC*, Δ*aceE*, Δ*metB*, and Δ*pepB* was increased, while that of Δ*gpx* and Δ*gor* was reduced. Importantly, the absence of *metB* abrogated the pyruvate potentiation and the loss of *gpx* or Δ*gor* distinctly promoted the effect (Fig. 6B). This *metB*-dependent abrogation implied that the flux of pyruvate to cysteine via CGL plays the most key role in the pyruvate potentiation, while *gpx*- or *gor*-dependent promotion suggested that the glutathione system dominates the potentiation. Thus, further experiments were performed in Δ*metB*, Δ*gpx*, and Δ*gor*. Intermediate metabolites, glycine, cysteine, and glutathione of the pyruvate–cysteine–glutathione system/glycine–ROS metabolic pathway promoted gentamicin killing in Δ*metB*, where glutathione caused similar killing to Δ*gpx* as pyruvate did (Fig. 6C). Moreover, ROS, GSH, GSSG, and GSH/GSSG were measured. Compared to EIB202, Δ*metB* exhibited a low abundance of ROS. When pyruvate was added, ROS was promoted, which was further boosted by the addition of gentamicin (Fig. 6D). To demonstrate that the killing in Δ*gpx* and Δ*gor* was attributed to the elevation of ROS, the ROS inhibitor α-tocopherol was used. Viability was elevated with increasing α-tocopherol dose in Δ*gpx* and Δ*gor*, when ROS was reduced (Fig. 6E and F). Absence of *gpx* increased GSH level but not GSSG and GSH/GSSG ratio, while absence of *gor* decreased GSH level, increased GSSG level, and decreased GSH/GSSG ratio. Deletion of *metB* caused down-regulation of GSH and GSSG, but increased the GSH/GSSG ratio. Similar results were detected in addition of gentamicin. When pyruvate or combination treatment with gentamicin was used, GSH was elevated, while GSSG expression and GSH/GSSG ratio were decreased in Δ*gpx* and Δ*metB.* However, a reversal result was measured in Δ*gor* (Fig. 6G)*.* The GSH/GSSG couple plays a crucial role in the 2-electron reduction of peroxides such as H_2_O_2_ using NADPH as an electron donor [29]. However, the GSH/GSSG ratio was not related to ROS expression level in Δ*gpx*, which exhibited higher ROS and a higher GSH/GSSG ratio. This is because the absence of *gpx* causes GSH accumulation and limits the GSSG source; thus, the accumulated GSH did not reduce ROS. Finally, a mouse model was used to confirm that the loss of *metB*, *gpx*, or *gor* leads to the changes in the resistance/sensitivity to gentamicin and gentamicin + pyruvate. Further, we observed 20% survival in EIB202-infected mice that had been treated with gentamicin. Subsequently, we also observed 40%, 30%, and 10% survival in mice infected with Δ*gpx*, Δ*gor*, and Δ*metB* mutants, respectively, following treatment with gentamicin alone (Fig. 6H). In the pyruvate combination treatment group, survival of Δ*gpx*, Δ*gor*, EIB202, and Δ*metB* was 90%, 70%, 50%, and 0%, respectively (Fig. 6I). Therefore, the pyruvate–cysteine–glutathione system/glycine–ROS metabolic pathway physiologically contributes to antibiotic resistance that can be reversed by exogenous pyruvate in vitro and in vivo.

### Role of the pyruvate–cysteine–glutathione system/glycine–ROS metabolic pathway in laboratory-evolved *E. tarda*

**Fig. 7.**
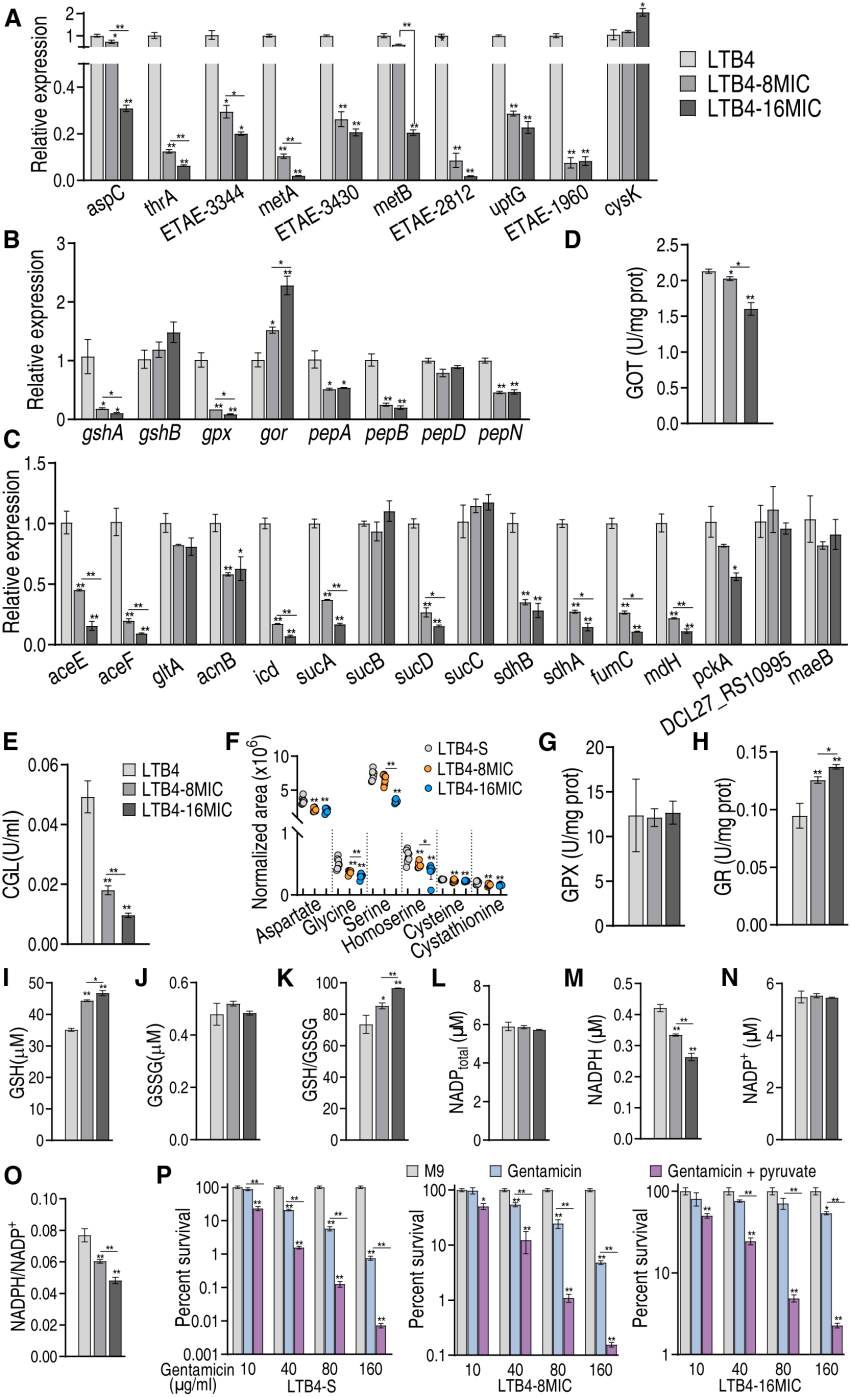
Detection of the pyruvate–glycine–glutathione system–ROS metabolic pathway in LTB4-R_8MIC_ and LTB4-R_16MIC_. (A to C) qRT-PCR for expression of genes encoding glycine, serine, and threonine metabolism, cysteine and methionine metabolism (A), glutathione metabolism (B), and the P cycle (C). (D and E) Activity of GOT (D) and CGL (E). (F) Abundance of some intermetabolites in glycine, serine, and threonine metabolism, cysteine and methionine metabolism, and glutathione system, quantified by GC-MS. (G and H) Activity of GPX and GR. (I to K) GSH (I), GSSG (J), and GSH/GSSG ratio (K). (L to O) NADP_total_ (L), NADP^+^ (M), NADPH (N), and NADP^+^/NADPH (O). (P) Survival of LTB4-S, LTB4-R_8MIC_, and LTB4-R_16MIC_ (10^6^ CFU/ml) in the presence of the indicated gentamicin with or without pyruvate. Results are displayed as the mean ± SD, and statistically significant differences are identified by Kruskal–Wallis followed by Dunn’s multiple comparison post hoc test unless otherwise indicated. *P < 0.05 and **P < 0.01.

We further explored whether the pyruvate–cysteine–glutathione system/glycine–ROS metabolic pathway is not only promoted by exogenous pyruvate but also inhibited during drug resistance. To do this, LTB4 and the 8- and 16-fold minimum inhibitory concentration (MIC) of gentamicin isogenic strains (LTB4-S, LTB4-R_8MIC_, and LTB4-R_16MIC_) were collected. Further, qRT-PCR showed that the reduced expression of most genes encoding the pyruvate to glutathione system via glycine, serine, and threonine metabolism, and cysteine and methionine metabolism was associated with an increasing MIC in LTB4-R_8MIC_ and LTB4-R_16MIC_ compared to LTB4-S (Fig. 7A to C). The activity of GOT (catalyzing the reversible reaction of oxaloacetate and l-glutamate into l-aspartate and α-ketoglutarate) and CGL (reversibly converting pyruvate to cysteine) was reduced in a MIC-dependent manner (Fig. 7D and E). Aspartate, glycine, serine, homoserine, cystathionine, and cysteine were decreased with increasing MICs (Fig. 7F). The activity of GPX and GR was either unchanged or up-regulated (Fig. 7G and H). Consistently, GSH and GSSG were increased or unchanged (Fig. 7I and J). The GSH/GSSG ratio was elevated with increasing MICs (Fig. 7K). Furthermore, NADP_total_, NAPDH, NAPD^+^, and NAPDH/NAPD^+^ ratios were measured. NADP_total_ and NAPD^+^ were unchanged, whereas NAPDH and NAPDH/NAPD^+^ ratios were reduced with the increasing MICs (Fig. 7L to O). Finally, pyruvate potentiation was demonstrated in these laboratory-evolved strains (Fig. 7P). These results indicate that the pyruvate–cysteine–glutathione system/glycine–ROS metabolic pathway contributes to antibiotic resistance.

### Role of the pyruvate–glycine–glutathione system–ROS metabolic pathway in other clinically isolated pathogens

**Fig. 8.**
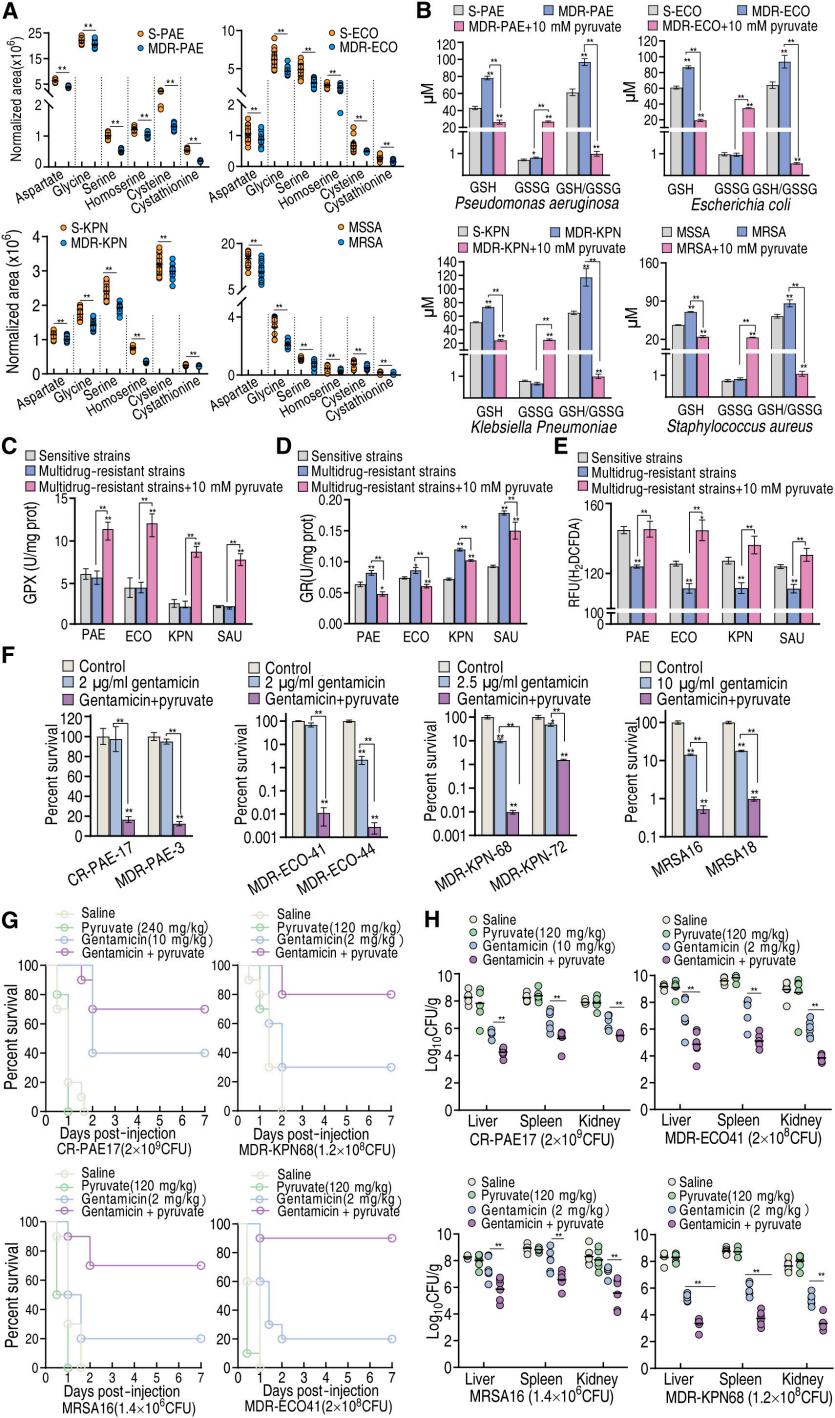
Pyruvate-potentiated killing and mechanisms in other clinical isolated pathogens. (A) Metabolomics of multidrug-resistant *P. auroginosa*, *E. coli*, *K. pneumonia*, and MRSA, showing changes in the pyruvate–aspartate–glycine–glutathione pathway. (B) GSH and GSSG and their ratio in 6 sensitive and 6 multidrug-resistant *P. auroginosa*, *E. coli*, *K. pneumonia*, and MRSA. (C and D) GPX (C) and GR (D) in 6 sensitive and 6 multidrug-resistant *P. auroginosa*, *E. coli*, *K. pneumonia*, and MRSA. (E) ROS in 6 sensitive and 6 multidrug-resistant *P. auroginosa*, *E. coli*, *K. pneumonia*, and MRSA. Strains were listed as follows: *P. auroginosa*, S-PAE643, S-PAE644, S-PAE645, S-PAE646, S-PAE647, S-PAE648, MDR-PAE3, MDR-PAE11, MDR-PAE12, MDR-PAE14, MDR-PAE16, CR-PAE17; *E. coli*, S-ECO61, S- ECO62, S-ECO63, S-ECO64, S-ECO65, S-ECO66, MDR-ECO41, MDR-ECO44, MDR-ECO45, MDR-ECO46, MDR-ECO47,MDR-ECO48; *K. pneumonia*, S-KPN100, S-KPN101, S-KPN102, S-KPN104, S-KPN106, S-KPN107, MDR-KPN68, MDR-KPN72, MDR-KPN76, MDR-KPN77, MDR-KPN78, MDR-KPN81; MRSA, MSSA1, MSSA2, MSSA3, MSSA4, MSSA5, MSSA6, MRSA11, MRSA12, MRSA13, MRSA14, MRSA16, MRSA18. Their MIC was listed in Fig. S7. (F) Survival of clinically isolated multidrug-resistant and/or carbapenem-resistant pathogens (10^8^ CFU/ml) in the presence or absence of 10 mM pyruvate and the indicated concentration of gentamicin. (G and H) Survival (G) and organ bacterial number (H) of mice systemically infected with the indicated pathogens in the absence and presence of the indicated concentration of pyruvate, gentamicin, or both. Results are displayed as the mean ± SD, and statistically significant differences are identified by Kruskal–Wallis followed by Dunn’s multiple comparison post hoc test unless otherwise indicated. *P < 0.05 and **P < 0.01.

To explore the role of the pyruvate–cysteine–glutathione system/glycine–ROS metabolic pathway in the resistance of other clinically isolated pathogens, clinically isolated multidrug-resistant and/or carbapenem-resistant *P. auroginosa*, *E. coli*, *K. pneumonia*, and MRSA were collected (Fig. S8). Metabolomics analysis revealed down-regulated aspartate, glycine, serine, homoserine, cysteine, and cystathionine in the pyruvate–cysteine–glutathione system/glycine–ROS metabolic pathway (Fig. 8A). Higher GSH, unchanged GSSG, and higher GSH/GSSG ratios were measured in 6 multidrug-resistant pathogens compared to 6 antibiotic-sensitive strains, which was reversed by exogenous pyruvate (Fig. 8B and Fig. S7). The activity of GPX was unchanged between these sensitive and resistant strains, which was promoted by exogenous pyruvate (Fig. 8C). However, GR activity was higher in these resistant strains compared to sensitive strains, and this activity was inhibited to low or normal levels by exogenous pyruvate compared to the sensitive strains (Fig. 8D). Moreover, lower ROS was consistently measured in resistant strains compared to sensitive pathogens, which was reversed by exogenous pyruvate (Fig. 8E). Exogenous pyruvate potentiated gentamicin to kill multidrug-resistant or carbapenem-resistant clinic isolates, *P. auroginosa*, *E. coli*, *K. pneumonia*, and MRSA (Fig. 8F). The pyruvate-potentiated gentamicin was effective in mouse models infected with these pathogens (Fig. 8G and H). Specifically, combination treatment with exogenous pyruvate and gentamicin increased survival in mice infected with MDR-ECO41, MDR-KPN68, CR-PAE17, and MRSA16 by 70%, 50%, 30%, and 50%, respectively, compared to gentamicin monotherapy (Fig. 8G). Plate counting in mouse livers, spleens, and kidneys showed that bacterial numbers were reduced by 241.9-, 268.8-, and 270-fold in mice infected with MDR-ECO41, 73.3-, 118.6-, and 43.1-fold in mice infected with MDR-KPN68, 23.5-, 31.3-, and 12.6-fold in mice infected with CR-PAE17, and 42.1-, 50.1-, and 12.2-fold in mice infected with MRSA16 (Fig. 8H). These results indicate that the pyruvate–cysteine–glutathione system/glycine–ROS metabolic pathway also confounds antibiotic sensitivity/resistance in these pathogens.

## Discussion

**Fig. 9.**
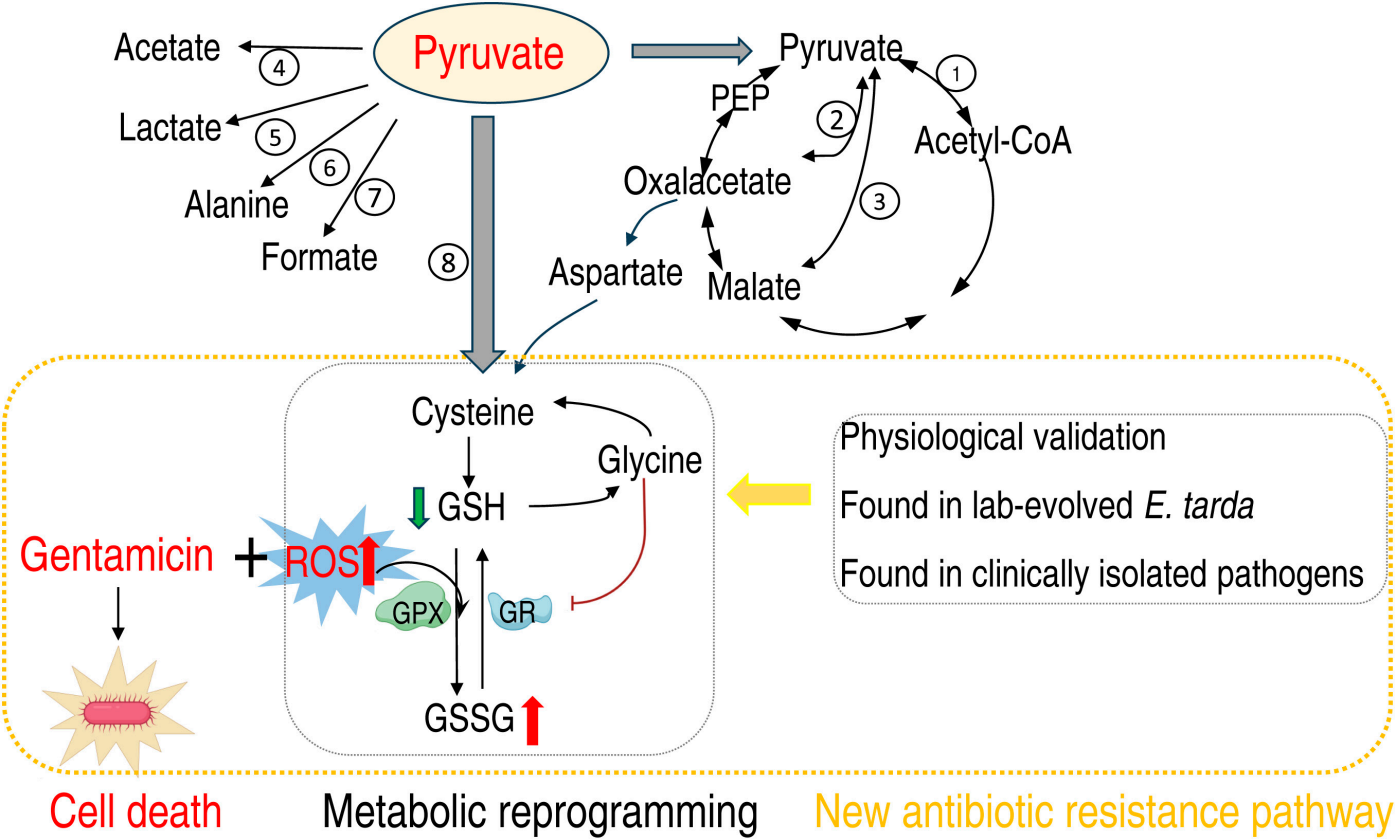
Proposed model. Pyruvate as a metabolic state reprogramming metabolite was used to potentiate gentamicin killing to multidrug-resistant *E. tarda*, which was involved in ROS dependence. This metabolic reprogramming leads to the finding on pyruvate–cysteine–glutathione system/glycine–ROS metabolic pathway. Specifically, the conversion of pyruvate to cysteine facilitates the synthesis of glutathione (GSH). The elevated GSH is subsequently metabolized into glycine to inhibit the activity of GR, thereby diminishing the availability of GSH. The decreased GSH reduces the GSSG/GSH ratio and thus compromises the capacity to scavenge intracellular ROS. The resulting ROS accumulation effectively potentiates the bactericidal effects of gentamicin. Furthermore, the metabolic pathway was physically demonstrated and determined as an antibiotic resistance mechanism in laboratory-evolved and other clinically isolated multidrug- and carbapenem-resistant pathogens.

Elucidating the mechanism of antibiotic resistance is an important prerequisite for controlling bacterial resistance [2]. The recently developed approach of metabolomic reprogramming has been documented to be effective at promoting antibiotic bactericidal efficiency [30–34], but whether this approach can be developed to reveal less well studied/neglected metabolic pathways that contribute to antibiotic resistance is unknown. The present study first demonstrates that pyruvate-mediated metabolic reprogramming reverses *E. tarda* resistance to gentamicin, thereby greatly promoting bactericidal efficiency in vitro and in vivo. Interestingly, this metabolic reprogramming is mostly dependent upon the boosting of the pyruvate–cysteine–glutathione system/glycine–ROS metabolic pathway to generate ROS that potentiate gentamicin killing. This metabolic pathway is a less studied pathway and was not previously known to be related to antibiotic resistance. The present study shows that this metabolic pathway not only physiologically contributes to antibiotic resistance but also plays a role in the biology of laboratory-evolved gentamicin-resistant *E. tarda* and clinically isolated multidrug-resistant and/or carbapenem-resistant *P. auroginosa*, *E. coli*, *K. pneumonia*, and MRSA (Fig. 9). Among the 8 pyruvate fluxes, only the other 6 and *pflB*, but not the one mediated by *metB*, have been discussed in recent reviews and references [21,22,35], although the *metB*-mediated pathway is indicated in the KEGG of bacteria, including the pathogens studied in the present study. Indeed, to the best of our knowledge, there is currently no research into the *metB*-mediated pathway. Therefore, the metabolic reprogramming approach can be used to reveal previously less studied metabolic pathways that contribute to antibiotic resistance. This finding highlights a novel way to explore previously unknown metabolic resistance mechanisms.

Pyruvate forms the center of the pyruvate node with PEP, playing an important role in the metabolism of enteric bacteria and other bacteria including *P. auroginosa*, *K. pneumonia*, and MRSA [36–40]. The metabolite is the terminal product from glycolysis and the starting source to the P cycle, which links the PEP–pyruvate–AcCoA pathway with the TCA cycle [17]. Pyruvate-induced/controlled metabolic flux to the P cycle/the TCA cycle and lactate, followed by the biosynthesis of fatty acid, glutamate metabolism, and purine metabolism, as well as the effect on electron transport chain have been extensively studied [12,17,41–46]. However, information regarding the metabolism of pyruvate to glycine, serine, and threonine metabolism, and cysteine and methionine metabolism, and then glutathione metabolism to regulate ROS is not available. The present study shows that exogenous pyruvate boosts glycine, serine, and threonine metabolism and cysteine and methionine metabolism via CGL and GOT. CGL converses pyruvate to cysteine directly, while GOT converts oxaloacetate to aspartate, where oxaloacetate comes from pyruvate by the intermetabolites, PEP and malate, by the P cycle indirectly and from pyruvate directly. Importantly, the PDH-mediated oxidation to acetyl-CoA fluxes to aspartate, which has traditionally been understudied. The boosting causes the disorder between GSH and GSSG, leading to depressed GSH and GSH/GSSG ratios, and a subsequent increase in ROS to enhance the bactericidal effect of gentamycin. Equally important, the boosting metabolic pathways are depressed in the laboratory-evolved and clinically isolated antibiotic-resistant bacteria. Comprehensive analysis on gene expression, enzyme activity, and gene mutation of CGL and GOT in pyruvate-boosting and antibiotic-resistant strains shows that CGL converts more pyruvate than GOT to influence the pyruvate–cysteine–glutathione system/glycine–ROS metabolic pathway. Nonetheless, our findings regarding that the absence of *metB* deprives the pyruvate potentiation suggests the importance of the previously neglected pyruvate flux. These findings indicate that the pyruvate–cysteine–glutathione system/glycine–ROS metabolic pathway is a previously unrecognized metabolic flux in antibiotic resistance, which can be modulated by the intermediate metabolites. Very importantly, the pyruvate–cysteine–glutathione system/glycine–ROS metabolic pathway provides a solution to overcome the antibiotic resistance mechanism of ROS down-regulation that is important in antibiotic-resistant bacteria.

Interestingly, the present study shows that the mechanism by which pyruvate promotes ROS is to increase glycine abundance leading to GR inhibition. Conversely, the down-regulation of glycine in antibiotic-resistant bacteria promotes the activity of GR, thereby reducing a large amount of GSSG to GSH, leading to ROS degradation. Many reports have shown that antibiotic-resistant bacteria exhibit deficiencies in glycine abundance [11,15,31,47,48], but the mechanism by which depression causes the elevated GSH to scavenge ROS is unknown. The regulation of an enzyme by a metabolite in order to manipulate ROS as a mechanism of antibiotic resistance has not previously been reported. Therefore, this finding highlights one method of elucidating the mechanism of antibiotic resistance from the perspective of metabolite regulation of enzyme activity.

The GSH-GSSG couple is the most abundant intracellular redox couple, playing a key role in antioxidant defense [24,49–52]. However, we show that GSH/GSSG ratio and ROS are not compatible in some cases. For example, GSH/GSSG level was increased in Δ*gpx* cells but ROS level was higher than WT cells. The absence of GPX deprives the oxidation of GSH to GSSG to degrade ROS, leading to up-regulation of GSH and ROS and down-regulation of GSSH, resulting in an elevated GSH/GSSG ratio. Therefore, when GSH/GSSG and ROS do not match, one potential explanation that needs to be ruled out is that these related catalytic enzymes are abnormally disturbed.

Whether antibiotics play a role via generating ROS and then ROS eliminates bacteria have been extensively studied [53,54]. However, the present study shows that ROS potentiate gentamicin killing. The potentiation is ROS concentration and gentamicin dependent. While it is clear that ROS down-regulation is an important feature of bacterial resistance, this finding suggests that ROS down-regulation in resistant bacteria is an important tactic for antibiotic resistance. It is also suggested that the molecules promoting ROS up-regulation may be antibiotic adjuvants. In contrast, pyruvate and ROS have a very delicate relationship. Pyruvate is regarded as a scavenger of chemical- and stress-induced (e.g., naringin-induced indole-3-carbinol, starvation, and low temperature) ROS in bacteria [28,55,56]. However, ROS elevation is measured during pretreatment by pyruvate [55]. A typical example is that *E. coli* pretreated with pyruvate exhibits elevated ROS, but scavenges subsequent ROS induced by indole-3-carbinol [55]. Thus, it seems that pyruvate has the dual role of promoting ROS production and eliminating ROS produced by some relevant conditions. This mechanism is revealed by studying this relationship along with ROS abundance and pyruvate concentration in the present study. However, the exact mechanism remains unclear.

Pyruvate is widely present in nature including cheese, apples, and red wine. Pyruvate is recommended as a dietary supplement for weight loss through reducing appetite. A daily dose of 5 to 30 g of pyruvate (salts) through oral administration for 3 to 6 weeks could reduce fatigue by increasing glucose uptake in skeletal muscles [57,58]. Rats injected intraperitoneally with sodium pyruvate (500 to 1,000 mg/kg) were still safe [59]. In this study, the dose of 240 mg/kg sodium pyruvate for in vivo administration is lower than the reported dose. Meanwhile, we did not observe any aberrant behaviors of mice under this does. Therefore, the combination of pyruvate and gentamicin has potential for clinical application, and metabolites like glycine or cysteine can be a replacement.

In summary, the present study shows that the metabolic reprogramming approach can be used to reveal antibiotic resistance mechanisms based on metabolic pathways, especially those that have not yet been fully characterized. Particularly, the pyruvate–cysteine–glutathione system/glycine–ROS metabolic pathway has been shown to contribute to antibiotic resistance, which was previously unknown because the direct flow of pyruvate to cysteine by *metB* from and the indirect flow of pyruvate to aspartate by *aspC* from oxaloacetate have not previously been studied in detail. Therefore, our data present a novel strategy to elucidate the metabolic mechanism behind antibiotic resistance based on the metabolic reprogramming approach. Moreover, the present study also provides an effective approach to combat antibiotic-resistant pathogens.

## Materials and Methods

### Source and culture conditions of bacterial strains

All bacterial strains came from the collection of our laboratory, where LTB4_-8MIC_ and LTB4_-16MIC_ originated from LTB4 that were sequentially propagated in tryptic soy broth (TSB) medium with ^1^/_2_ MIC of gentamicin. *E. tarda* strains were cultured in fresh TSB medium at 30 °C, with shaking at 200 rpm for 24 h, while the other bacteria were cultured in fresh Luria–Bertani medium (LB medium) at 37 °C, with shaking at 200 rpm for 16 h. The overnight bacterial cultures were collected, washed twice with saline, and resuspended in an M9 medium (containing 17.1 g/l Na_2_HPO_4_·12H_2_O, 3 g/l KH_2_HPO_4_, 1 g/l NH_4_Cl, 0.5 g/l NaCl) to an optical density at 600 nm (OD_600_) of 0.2 when metabolites and/or antibiotics were added if desired; 10 mM sodium acetate, 2 mM 7H_2_O·MgSO_4_, and 0.1 M CaCl_2_ were supplemented. Metabolites were added if desired. The bacterial cells were then cultured at 37 or 30 °C with shaking at 200 rpm for 6 h, excluding the incubation period experiment.

### Measurement of MIC

MICs were tested using Clinical and Laboratory Standards Institute (CLSI) methods. Overnight LB cultures were diluted and grown to OD_600_ = 0.5, and then 10 μl of 5 × 10^6^ colony-forming units (CFU)/ml bacteria was mixed with 100 μl of diluted antibiotics in a 96-well plate and incubated for 16 h at 37 °C. The MIC was the lowest concentration preventing growth.

### Sample preparation for GC-MS analysis

Sample preparation for the metabolomes of PPD200/87 and PPD200/87 plus pyruvate was carried out as previously described [60]. Bacterial cultures were grown in TSB medium at 30 °C and 200 rpm for 24 h, diluted 1:100 into fresh TSB, and cultured until OD_600_ reached 1.0. The cells were harvested, washed with sterile saline, and quenched with precooled methanol. After sonication (650 W, 35% output, 2 s on, 3 s off) and addition of ribitol as an internal standard, the mixture was centrifuged to obtain supernatants, which were dried under vacuum at 37 °C. The dried samples were then derivatized with methoximation–pyridine hydrochloride for 3 h at 37 °C, followed by *N*-methyl-*N*-trimethylsilyltrifluoroacetamide (MSTFA) treatment for 30 min at the same temperature.

### Metabolomic data analysis

GC-MS was performed in Agilent 7890A GC equipped with an Agilent 5975C VL MSD detector (Agilent Technologies). The sample of 1 μl was injected into a 30 m × 250 μm internal diameter (i.d.) × 0.25 μm DB-1ms GC column. Initial temperature of the GC oven was kept at 85 °C for 5 min followed by an increase to 270 °C at a rate of 15 °C/min and then held for 5 min. Helium was used as the carrier gas, and flow was kept constant at 1 ml/min. The MS was operated in a range of 50 to 600 m/z. Each sample was analyzed in 4 biological repeats with 2 technical replicas. Identification of metabolites corresponding to the chromatographic peaks in the GC-MS analysis was performed using the National Institute of Standards and Technology (NIST) Mass Spectral Library. The data were normalized by applying total amount correction and generating standardized datasets consisting of metabolite information, retention times, and peak areas, which were further processed for metabolomics analysis. Significant differences in the standardized data were calculated and selected using IBM SPSS Statistics 19 software, considering a threshold of *P* < 0.01. Cluster analysis was performed using R software (R×64 3.6.1). The normalized areas of differential metabolites were analyzed using *z* score. Principal components and S-plot analyses were carried out using SIMCA-P+12.0 software (version 12; Umetrics, Umea, Sweden), while metabolic pathway analysis was performed using MetaboAnalyst 4.0 enrichment. Figures were created using GraphPad Prism 8.0 and Adobe Illustrator CS6.

### RNA isolation and transcriptome sequencing

Bacterial collection was performed according to the method described in metabolomic profiling. Total RNA was extracted from 3 ml of bacterial cultures using the TRIzol Reagent Kit (Thermo Fisher Scientific, MA, USA), as previously reported [61].

### Quantitative real-time polymerase chain reaction

To investigate the effect of gentamicin and pyruvate on gene expression levels, qRT-PCR was conducted following previously described methods [62]. Primers used in this study are listed in Table S1.

### Enzyme activity assay

Bacterial cells were cultured in M9 medium with/without metabolites at 30 °C and 200 rpm for 6 h. After the incubation, cells were centrifuged at 8,000*g* for 5 min, washed with phosphate-buffered saline (PBS), and adjusted to OD_600_ = 1.0. The cells were sonicated in PBS for 10 min at 650 W × 35% power (2 s on, 3 s off) and centrifuged at 12,000*g* and 4 °C for 10 min to collect supernatants. Protein quantification was conducted using the BCA Protein Assay Kit. Enzyme activities were measured using specific assay kits (GOT: Solarbio BC1565, China, CGL: HalingBio HL50550.1v.A, China, GPX: Solarbio BC1195, China, and GR: Solarbio BC 1165, China) and colorimetric readings at designated wavelengths.

### Antibiotic bactericidal assay

In accordance with previous reports [12], antibiotic killing assays involved centrifuging samples at 8,000 rpm for 3 min, washing with 50 ml of sterile saline, and resuspending in M9 medium with supplements to OD_600_ = 0.2. Antibiotics were added, and samples were incubated at 30 °C for 6 h. Survival was assessed by periodic sampling, serial dilution, and CFU counting on TSB plates after 24 h at 30 °C. Survival rates were calculated relative to controls, with at least 3 biological replicates.

### Biofilm cultivation

The biofilm cultivation experiment was conducted according to previously published methods [12]. In brief, PE50 catheters measuring 6 mm in length and 0.58 mm × 0.96 mm in dimeter were utilized. The catheters were inoculated with 1 ml of LB medium containing 10 μl of overnight cultures and incubated aerobically at 37 °C for 24 h. The medium was replaced by fresh LB every 24 h for 3 d. To remove loosely adherent cells, the PE50 catheters were washed 5 times with 1 ml of saline.

### Bacterial persister assay

The persister cultivation experiment was conducted according to a previously published method [8]. PPD200/87 was cultured overnight to saturation, then washed with saline, and centrifuged at 8,000 rpm for 5 min. The samples were resuspended in TSB to OD_600_ = 1.0 and exposed to escalating ofloxacin concentrations for 4 h to identify persisters. These persisters were then washed, resuspended in M9 to OD_600_ = 0.2, and treated with gentamicin and sodium pyruvate. After 6 h at 30 °C and 200 rpm, colonies were counted as before.

### Sodium pyruvate-enabled killing of pathogens by gentamicin in a mouse model

The experiment was performed as previously described [63]. Balb/c mice (18 to 20 g) from Sun Yat-sen University’s Animal Center were acclimated for a week before use in pathogen-killing and survival studies. Mice were infected intraperitoneally with multidrug-resistant strains and treated intramuscularly 1 h later with saline (group 1), sodium pyruvate (group 2), gentamicin (group 3), or both (group 4). Organs were harvested at 6 h for CFU/g analysis. For survival tests, 20 mice per group were infected and monitored for 7 d.

### Sodium pyruvate-enabled killing of pathogens by gentamicin in a tilapia model

Three-centimeter-long, 0.4-g tilapia (*Oreochromis mossambicus*) from a Guangzhou breeder, confirmed infection-free, were acclimated for 2 weeks at 25 °C in a recirculating aquarium system with a basal diet. Eighty tilapia were split into 4 groups of 20 for intraperitoneal bacterial infection. One hour later, they were injected with 5 μl of saline (control), 30 μg of gentamicin, 90 μg of sodium pyruvate, or both. Survival was tracked for 7 d.

### Measurement of total GSH, GSH, and GSSG

The GSSG/GSH Quantification Kit II (DOJINDO, Japan) was used for these experiments, where bacteria were cultured in M9 medium with various treatments at 30 °C for 6 h, adjusted to OD_600_ = 1.0, and sonicated. Protein content was measured with a BCA kit (Beyotime, China), and absorbance at 405 nm was read using a microplate reader (BioTek Instruments, USA), with data normalized to protein levels.

### Measurement of redox status

Bacteria in M9 with additives were cultured at 30 °C for 6 h, mixed with 2′,7′-dichlorofluorescin diacetate (Sigma), and incubated at 37 °C for 30 min. Fluorescence was read at 485 nm/535 nm on a Victor X5 plate reader.

### Quantification of NADPH level

NADPH level was determined using a NADP^+^/NADPH Assay Kit with WST-8 (Beyotime, S0179, China) following the manufacturer’s instructions.

### Quantification of intracellular gentamicin

Bacteria at OD_600_ = 0.2 were cultured with pyruvate/H_2_O_2_ and gentamicin at 30 °C for 6 h, washed with PBS, and resuspended to OD_600_ = 1.0. Gentamicin concentration was measured using a Gentamicin ELISA Kit (Shanghai Jianglai Industrial Limited By Share Ltd., JL17568, China) after sonication.

### Gene knockout

A gene deletion mutant was constructed from *E. tarda* EIB202 via *sacB*-based allelic exchange [54]. Briefly, using the genome of *E. tarda* EIB202 as the template, primers were set at about 500 base pairs (bp) up- and downstream of the gene to be knocked out and at appropriate positions within the gene (Table S3). A DNA fragment of ~1,000 bp in length with no target gene was obtained following 2 PCRs. This DNA fragment was seamlessly cloned into the linearized pDS132 vector, and the product was transferred into *E. coli* MC1061 to screen for positive clones (survival in kanamycin containing medium). The recombinant plasmid was further transformed into *E. coli* MFD-λpir (survived in medium containing 2,6-diaminopeptic acid) and screened for positive clones. All positive clones were verified by PCR (primers as above). *E. coli* MFD-λpir and *E. tarda* EIB202 were mixed at a ratio of 4:1 and cultured on TSB solid medium overnight at 30 °C for conjugation transfer. Then, the bacteria were washed with sterile saline and the mixture was collected, which was coated on TSB solid medium (containing 50 μg/ml kanamycin, 30 °C, 24 h). The positive clones were selected from the plate and cultured in 1ml of fresh TSB medium at 30 °C and 200 rpm. After about 24 h, the bacterial solution was coated on TSB solid medium (containing 12.5% w/v sucrose, 30 °C, 36 h). Then, the colonies without kanamycin resistance were selected from the plates and further verified by PCR and sequencing. Primers used to construct deletion mutants are listed in Table S2.

### Cloning, expression, and purification of GR gene/protein

Cloning of GR gene and expression and purification of GR recombinant proteins were performed using a routine procedure as previously described [64]. Primers were designed for PCR amplification (Table S3). pET-32a and *E. coli* BL21 were used as expressional vector and cells.

### Isothermal titration calorimetry

ITC (Nano-ITC, TA Instruments, USA) was used to detect the thermodynamic parameters between GR with glycine at 25 °C. The detection procedure was carried out as the same as previously described [65].

### Ethics statement

Animal work was conducted in strict accordance with the recommendations in the Guide for the Care and Use of Laboratory Animals of the National Institutes of Health. The protocol was approved by the Institutional Animal Care and Use Committee of Sun Yat-sen University (approval no. SYSU-IACUC-2020-B126716).

## Data Availability

The RNA-sequencing data were deposited to the National Center for Biotechnology Information with accession number PRJNA1110554.
