# Supplementary material for: Pyruvate Abundance Confounds Aminoglycoside Killing of Multidrug-Resistant Bacteria via Glutathione Metabolism
Source: Research (Wash D C). 2024 Dec 18;7:0554. doi: 10.34133/research.0554 (PMC11654824; doi:10.34133/research.0554)
Supplement: Supplementary 1 — Figs. S1 to S8 Tables S1 to S3 [file research.0554.f1.pdf]

Supplementary Materials for

Pyruvate Abundance Confound Aminoglycoside Killing of Multidrug-resistant Bacteria via Glutathione Metabolism

Jiao Xiang [1,2], Si-qi Tian [1, 2], Shi-wen Wang [1] , Ying-li Liu [1,2], Hui Li [1,2], Bo Peng[1,2, *]

*Corresponding author. Email: pengb26@mail.sysu.edu.cn

This PDF file includes:

Figures S1 to S8

Tables S1 to S3

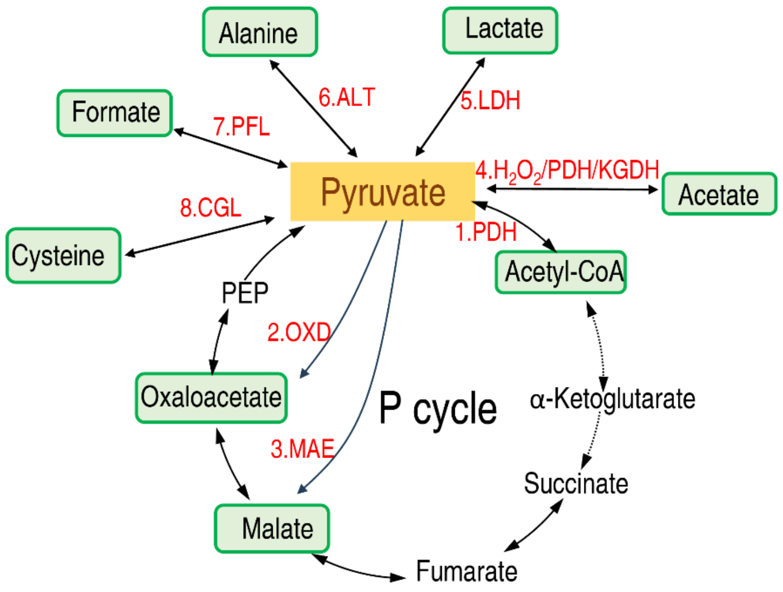

figure S1 Pyruvate has seven metabolic fates

Antibiotics	μg/mL
Sulfamethazine	125
Sulfamethoxazole	125
Roxithromycin	50
Colistin	125
Sulfamonomethoxine	7.81
Sulfadiazine	7.81
Tetracycline	50
Doxycycline	31.25
Thiamphenicol	62.5
Neomycin	7.81
Flufenicol	<1.95
Fluoroquine	<1.95
Gentamicin	6.25

Fig. S2 MIC of PPD200/87

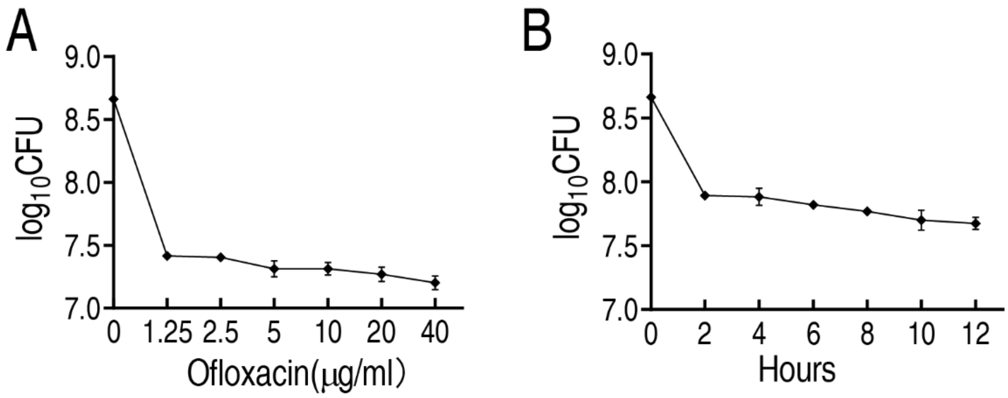

Fig. S3 Preparing of PDD200/87 pesisters. (A) Survival of PDD200/87 in the indicated concentration of ofloxacin. **(B)** Survival of PDD200/87 in the indicated incubation time.

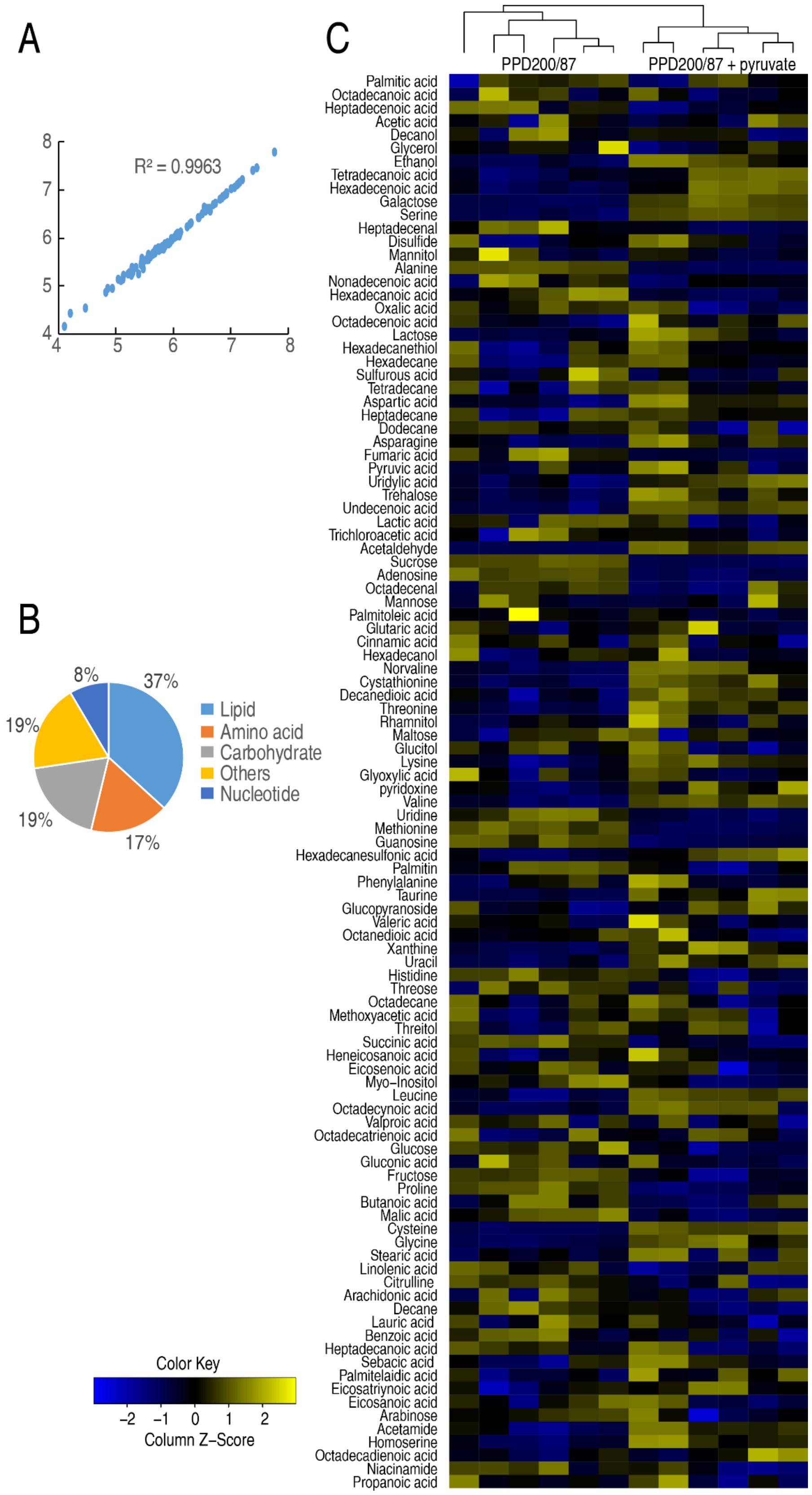

Fig. S4 Metabolic profile of PPD200/87 in medium with and without pyruvate.

(A) Abundance correlation of metabolites quantified in two technical replicates with high reproducibility. Metabolite abundance was represented in $\log_{10}$.

(B) Category of metabolites detected.

(C) Heatmaps of unsupervised hierarchical clustering showing differential abundance of metabolites of PPD200/87 in medium with and without pyruvate.

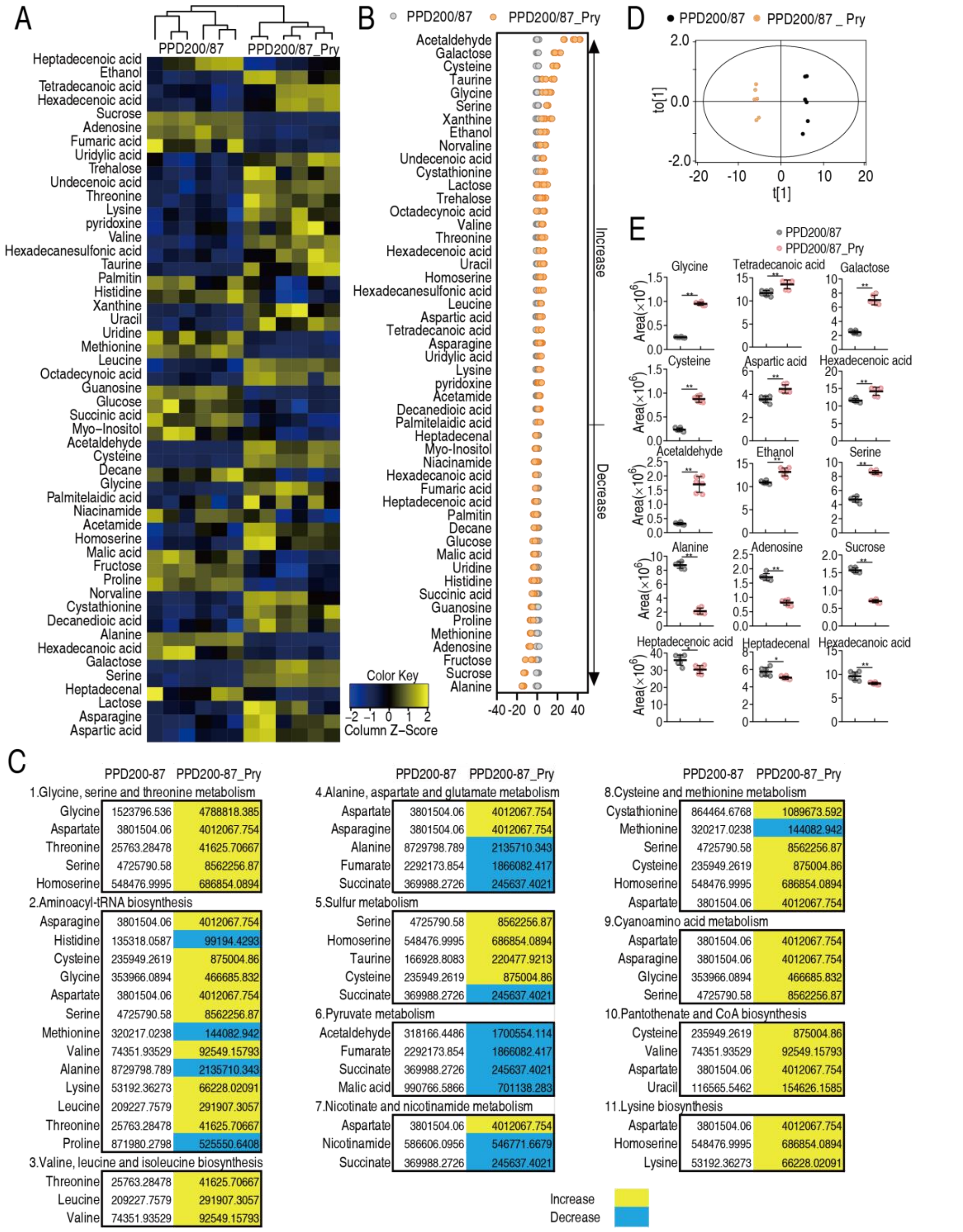

fig. S5 Differntial metabolome and biomarkers

(A) Heat map showing differentially abundant metabolites between PPD200/87 and PPD200/87+pyruvate. Yellow and blue colors indicate increase and decrease of the metabolites scaled to mean and standard deviation of row metabolite level, respectively (see color scale).

(B) Z-score plot of differentially abundant metabolites. The data were separately scaled to the mean and SD of the controls. Each point represents one metabolite in one technical repeat and colored by sample types.

(C) Integrative analysis of metabolites in enriched metabolic pathways.

(D) OPLS-DA analysis.
(E) Abundance of biomarkers.

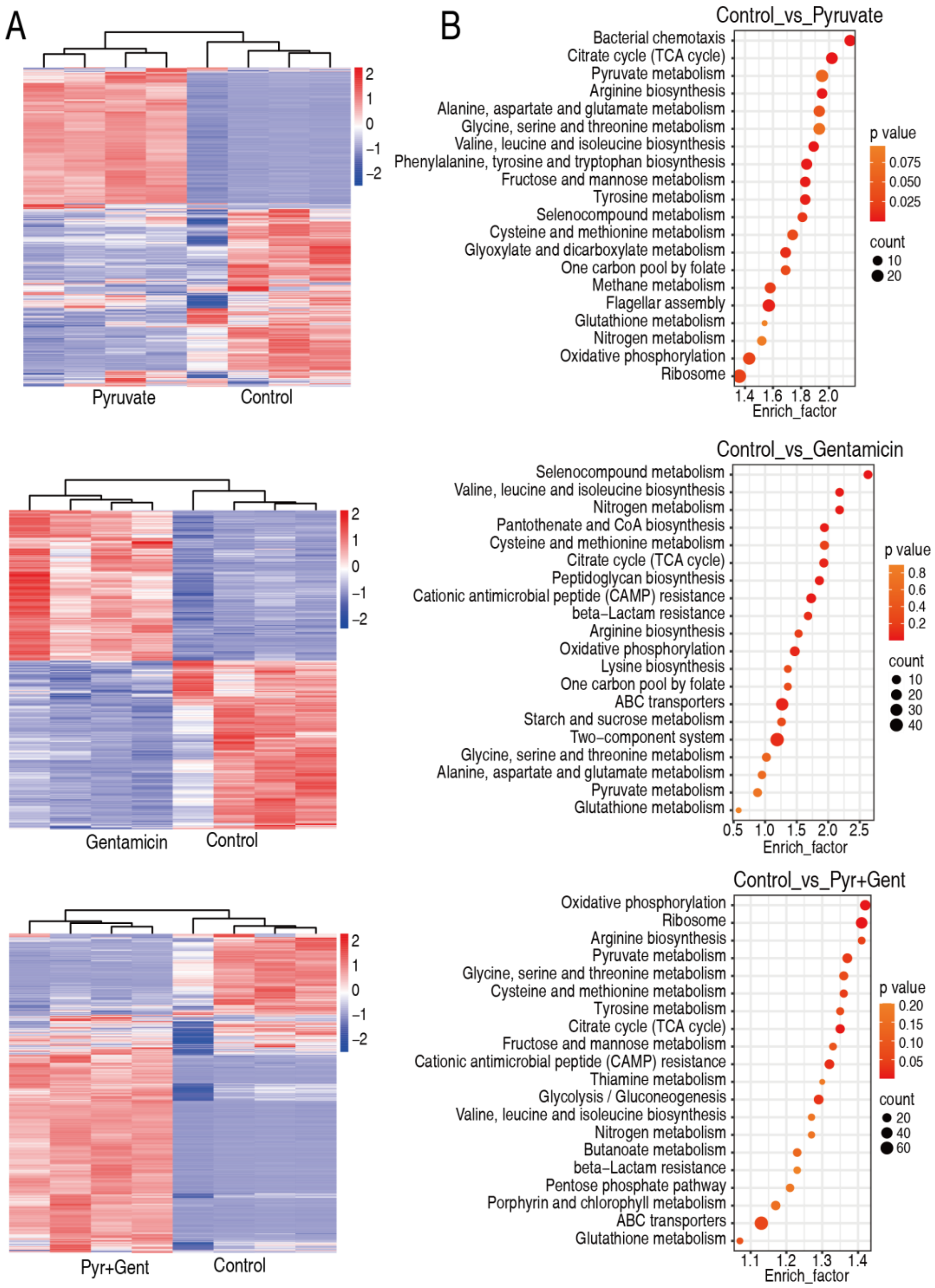

Fig. S6 Transcriptome analysis for DEGs and pathway enrichment.

(A) Heat map showing DEGs between PPD200/87, PPD200/87+pyruvate, PPD200/87+gentamicin, PPD200/87+pyruvate+gentamicin. Red and purple colors indicate increase and decrease of the metabolites scaled to mean and standard deviation of row metabolite level, respectively (see color scale).

(B) Pathway enrichment.

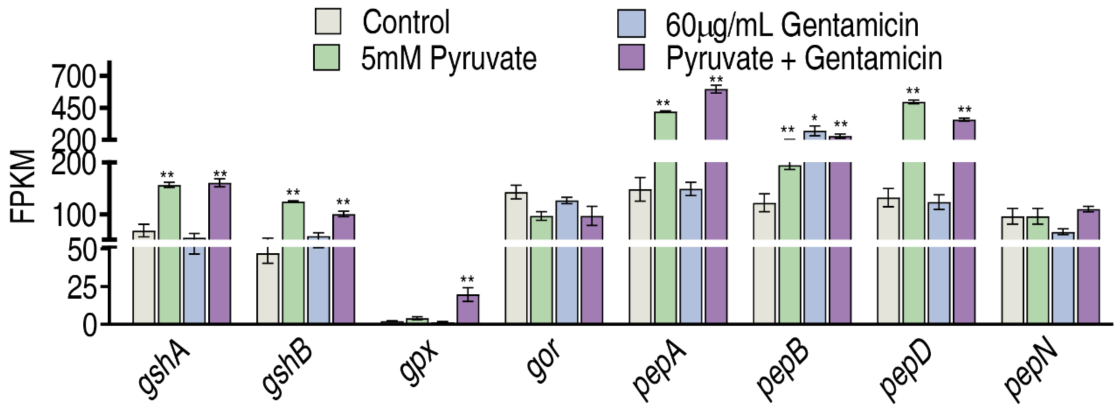

fig. S7 Transcriptome analysis for these indicated genes

	Ampicillin	Cefoperazone	Ceftazidime	MeropeneM	Gentamicin	Tobramycin	Kanamycin	Levofloxacin	Ofloxacin	Ciprofloxacin
S-KPN100	128	0.0625	0.25	0.031	4	2	2	0.0625	0.0625	0.031
S-KPN101	128	0.0625	1	0.031	2	2	2	0.0625	0.0625	0.031
S-KPN102	128	0.0625	0.25	0.031	2	2	4	0.0625	0.0625	0.031
S-KPN104	128	0.0625	0.125	0.031	2	2	2	0.0625	0.0031	0.031
S-KPN106	128	0.25	0.5	0.031	2	2	2	0.0625	0.0625	0.031
S-KPN107	128	0.25	0.0625	0.031	2	2	2	0.0625	0.0625	0.031
MDR-KPN68	⩾256	⩾256	32	0.0625	8	16	8	2	8	8
MDR-KPN72	⩾256	⩾256	2	0.0625	8	16	4	8	0.25	2
MDR-KPN76	⩾256	⩾256	64	0.0156	64	32	16	16	8	8
MDR-KPN77	⩾256	⩾256	128	0.0625	64	32	8	16	8	8
MDR-KPN78	⩾256	⩾256	0.25	0.0156	32	4	16	16	16	16
MDR-KPN81	⩾256	⩾256	1	0.0625	32	2	16	32	4	32
S-PAE643	32	4	2	1	0.25	1	2	0.25	1	0.25
S-PAE644	32	8	2	1	0.125	0.5	1	0.25	2	0.25
S-PAE645	32	2	2	0.25	0.5	1	0.5	1	1	0.125
S-PAE646	64	2	2	0.5	1	1	0.25	0.5	2	0.0625
S-PAE647	16	4	2	0.25	0.25	0.25	0.5	0.5	1	0.25
S-PAE648	16	2	2	0.25	0.25	0.25	0.5	0.25	2	0.125
MDR-PAE16	⩾256	⩾256	8	1	16	4	64	16	8	1
MDR-PAE12	⩾256	⩾256	2	2	16	8	64	8	8	0.5
MDR-PAE11	⩾256	⩾256	2	4	64	6	64	8	4	0.25
MDR-PAE14	⩾256	⩾256	4	2	32	2	64	16	2	2
MDR-PAE3	⩾256	⩾256	2	0.5	16	2	128	8	1	0.125
CR-PAE17	⩾256	⩾256	2	8	16	32	128	8	4	2
MSSA1	4	4	4	0.25	2	2	2	0.5	1	0.125
MSSA2	2	2	2	0.5	2	2	4	1	1	0.031
MSSA3	4	1	4	1	1	0.5	0.25	2	2	0.031
MSSA4	8	2	1	0.25	0.25	0.5	0.5	0.25	1	0.031
MSSA5	4	1	4	0.5	0.5	1	5	0.5	0.25	0.0625
MSSA6	8	0.5	2	1	2	2	1	1	0.5	0.125
MRSA11	⩾256	⩾256	64	2	32	16	16	16	8	2
MRSA12	⩾256	64	32	4	32	16	32	8	16	2
MRSA13	⩾256	64	64	2	16	8	16	16	32	0.125
MRSA14	⩾256	32	64	2	32	16	16	16	16	2
MRSA16	⩾256	⩾256	128	16	16	4	16	16	32	0.25
MRSA18	128	16	64	2	16	2	16	4	8	0.125
S-ECO61	2	1	2	0.0625	0.5	0.5	0.25	0.25	0.5	0.25
S-ECO62	1	0.25	0.5	0.0625	2	0.25	0.125	0.25	0.5	0.125
S-ECO63	0.25	0.25	1	0.031	0.5	0.125	0.5	0.5	0.125	0.0625
S-ECO64	1	0.125	0.5	0.031	0.5	0.25	1	1	0.25	0.25
S-ECO65	0.5	1	0.125	0.0156	0.5	0.5	1	0.25	1	0.125
S-ECO66	0.5	0.5	0.5	0.0625	2	1	0.5	0.125	0.25	0.125
MDR-ECO41	⩾256	⩾256	16	0.5	8	16	32	16	1	0.25
MDR-ECO44	⩾256	⩾256	4	0.5	8	16	2	8	0.5	0.125
MDR-ECO45	⩾256	⩾256	16	1	16	8	32	8	2	0.25
MDR-ECO46	⩾256	⩾256	8	0.5	8	16	16	32	2	0.5
MDR-ECO47	⩾256	⩾256	16	2	16	16	8	16	0.5	1
MDR-ECO48	⩾256	⩾256	16	1	16	8	2	8	1	0.5

Fig. S8 MIC of clinically isolated multidrug-resistant (MDR) and carbapenem-resistant pathogens.

Table S1 Primers for qRT-PCR

Gene		Sequence (5'-3')	Gene		Sequence (5'-3')
ETAE_16S	Forward	ACTGAGACACGGTCCAGACTCCTAC	*sucA*	Forward	GCGTTTCATCATCTGACGCC
	Reverse	TTAACGTTCACACCTTCCTCCCTAC		Reverse	TTCTCGTCGGTGTTGGTCAG
aspC	Forward	GTATCTGGGTGAGCAACCCC	*sucB*	Forward	CCTGATGTCCACGCCGATTA
	Reverse	TTATGGCAACAGCCGTGGAA		Reverse	ATGGTCATACGAAAGCGCCA
thrA	Forward	AGCGGTATTTCCGTGGTGTT	*sucD*	Forward	TATGAGGCGGTGAAGCAGAC
	Reverse	TCGATAACATCCAGCGGCTC		Reverse	TCACCGATCATCACGATGGC
ETAE_3344	Forward	TGTCAGGGCGGCGATTATAC	*sucC*	Forward	GCAATATCGGCTGCATGGTG
	Reverse	GGTTTTCACTCCGCTCTCCA		Reverse	CCGCCGAAAATGTTGACCAG
metA	Forward	GGCTTTGACGACGGCTTTTT	*sdhB*	Forward	GTTACAACCCGGACGTGGAT
	Reverse	CATACTCCGGATGGCCTGTC		Reverse	CCGTTTTGGCCGTTCATGTT
ETAE_3340	Forward	TGCTGCACTCCTGTACCAAG	*sdhA*	Forward	AGTCAGAGAGTTTGACGCCG
	Reverse	CGCCGGTCACACCGATATTA		Reverse	GAAACGGTATGGGAACGGGT
metB	Forward	TGATATCGTCGTGCACTCGG	*fumC*	Forward	AATGTCTTTCGTCCGCTGGT
	Reverse	TTGTGCAGATAGGCCAGACG		Reverse	GCAGCAGTTCATCGATACGC
ETAE_2812	Forward	CACCACCACCCATAAGACCC	*mdH*	Forward	CGGCATCATCCGTAACCTGA
	Reverse	CTGCTGCTGGTAACGGGTAA		Reverse	GAAGGTGTTGGAGCGGATGA
uptG	Forward	CGGACAAGATGAGCGGAGAG	*pckA*	Forward	AAGGCTTAACCGCAGCTGAT
	Reverse	GGTCGATATACAGGCTGCCC		Reverse	GTAACGGTACCGCGCTCATA
ETAE_1960	Forward	ATCCTTCGCTACATCCGCAG	*MaeB*	Forward	CCGGAGTTGGAGATCGATGG
	Reverse	GCAGCCATACGCTGAACTTG		Reverse	AATATTGGCGGCCCCTTTCA
DCL27_RS10995	Forward	AGAGCCGCCTCGTACAAAAA	*gshA*	Forward	TTTCTGATCTGGTGCGTGCT
	Reverse	GCATTTCGCGAATGATCGCT		Reverse	CATGACAGCCAATGCCGATG
cysK	Forward	CGGACCGCACAAAATCCAAG	*gshB*	Forward	CAGGATGACCCCAACCTGTC
	Reverse	CGAGGATACCCTCTTCACGC		Reverse	GTTTCGCCGCTTTTGGGAAT
aceE	Forward	GAAACTGTTTGCCGAGCAGG	*gpx*	Forward	ACCTTTCATACCCGCCAAGG
	Reverse	GTGGTGACGCAGGTTTTCAC		Reverse	GGCAAGTGGCTAGAAGAGCA
aceF	Forward	CCGGAGACAAGGTGAAGACC	*gor*	Forward	CCGGGCGCAGAATATGGTAT
	Reverse	CAACCTCATCACCGCCGATA		Reverse	ACGGACAAACAGGTGGGTTT
gltA	Forward	GCGCGCCAAGGATAAGAATG	*pepA*	Forward	ATGCCGCCCAACATCTGTAA
	Reverse	GCTACCTCCAGCAGATCGTC		Reverse	ATCACCGACATCAGCGACTC
acnB	Forward	GGGATATACCCTGGCGCAAA	*pepB*	Forward	AGGGGATGAAGCAGGTTTCG
	Reverse	GCAGGCCAGATCCTTCAACT		Reverse	TATTCAGCAGCCGACGATCC
icd	Forward	TGATCAAGGTGGTGGATGCC	*pepD*	Forward	TGTGGCAGATCTTTGCCGAT
	Reverse	ATAGACCTGGGTCGCCTTCT		Reverse	CACCACCGGAATGCGTTTTT
			pepN	Forward	GATTCATCCGGCGCAAAACA
				Reverse	TTTATCCGCCACGATACGGG

Table S2 Primers for construction of gene-deleted mutants

Primer	Sequence (5'-3')
pepB-For	ggttaaaaaggatcgatcctGAAGGCTTTGACTCCCTTGGGG
pepB-int-Rev	CCTGCTTCATCCCCTGGGTATCCA
pepB-int-For	TACCCAGGGGATGAAGCAGGTCCTGTCGCACTTCGTCACTGACTA
pepB-Rev	tcgatatcgcatgcggtaccTTTAAAAACAGCGTCTCGCCGC
gor-For	gggttaaaaaggatcgatcctACCTATGCACTGTGGTACCCGG
gor-int-Rev	CGATTGATGGAGGCGATACCGCC
gor-int-For	GGTATCGCCTCCATCAATCGGATCCACGGGATCGGCTTCGG
gor-Rev	tcgatatcgcatgcggtaccTACGTAGCCTGGCTCAGCGTAG
metB-For	GGTTAAAAAGGATCGATCCTCTAACCCGGCCAACCCCGC
metB-int-Rev	GGGGTTGCCGCTACGGGCGTATTC
metB-int-For	CCCGTAGCGGCAACCCCTGCGTGCTGCGCGGCGATG
metB-Rev	TCGATATCGCATGCGGTACCAGAAGCGCACCATTCTGAT
aspC-For	GGTTAAAAAGGATCGATCCTAAGTACGACGCCAACAACGT
aspC-int-Rev	TTTAACGCTGGTCAGCACCG
aspC-int-For	TGCTGACCAGCGTTAAAGGCGTCTACGCCGTCAGCT
aspC-Rev	TCGATATCGCATGCGGTACCGGTGCTCGGGGAGAGCGTGA
gpx-For	GGTTAAAAAGGATCGATCCTGGGGAATGCCTGGTGCAGCA
gpx-int-Rev	ACTCGCGGAACAGCGCGTCGCTGGTG
gpx-int-For	CGACGCGCTGTTCCGCGAGTATGAAGAGATCATCCTGGGTA
gpx-Rev	TCGATATCGCATGCGGTACCATGCGTTTGGCATGGATACG

Table S3 Primer for gene clone

**Primer**	**Sequence (5’-3’)**
gor-For	gctgatatcggatccgaattcatgactaaacattatgactatctcgc
gor-Rev	TTAGATCCTCCTCCTGCGGCCGCttaacgcatggtgacaaactcc
